# Remodeling tumor‐associated macrophage for anti‐cancer effects by rational design of irreversible inhibition of mitogen‐activated protein kinase‐activated protein kinase 2

**DOI:** 10.1002/mco2.634

**Published:** 2024-07-10

**Authors:** Danyi Wang, Deqiao Sun, Xiaoyan Wang, Xia Peng, Yinchun Ji, Lu Tang, Qichang He, Danqi Chen, Ye Yang, Xuan Zhou, Bing Xiong, Jing Ai

**Affiliations:** ^1^ State Key Laboratory of Drug Research Shanghai Institute of Materia Medica Chinese Academy of Sciences Shanghai P. R. China; ^2^ School of Pharmacy University of Chinese Academy of Sciences Beijing P. R. China; ^3^ State Key Laboratory of Chemical Biology Shanghai Institute of Materia Medica Chinese Academy of Sciences Shanghai P. R. China; ^4^ Shandong Laboratory of Yantai Drug Discovery Bohai Rim Advanced Research Institute for Drug Discovery Yantai P. R. China

**Keywords:** anti‐tumor, irreversible inhibitor, kinase, macrophage, mitogen‐activated protein kinase‐activated protein kinase 2 (MK2)

## Abstract

Mitogen‐activated protein kinase‐activated protein kinase 2 (MK2) emerges as a pivotal target in developing anti‐cancer therapies. The limitations of ATP‐competitive inhibitors, due to insufficient potency and selectivity, underscore the urgent need for a covalent irreversible MK2 inhibitor. Our initial analyses of The Cancer Genome Atlas database revealed MK2's overexpression across various cancer types, especially those characterized by inflammation, linking it to poor prognosis and highlighting its significance. Investigating MK2's kinase domain led to the identification of a unique cysteine residue, enabling the creation of targeted covalent inhibitors. Compound **11** was developed, demonstrating robust MK2 inhibition (IC_50 _= 2.3 nM) and high selectivity. It binds irreversibly to MK2, achieving prolonged signal suppression and reducing pathological inflammatory cytokines in macrophages. Furthermore, compound **11** or MK2 knockdown can inhibit the tumor‐promoting macrophage M2 phenotype in vitro and in vivo. In macrophage‐rich tumor model, compound **11** notably slowed growth in a dose‐dependent manner. These findings support MK2 as a promising anticancer target, especially relevant in cancers fueled by inflammation or dominated by macrophages, and provide compound **11** serving as an invaluable chemical tool for exploring MK2's functions.

## Graphical abstract

Through rational design covalent MK2 inhibitors targeting the unique cysteine residue, we discovered that compound **11** exhibited potent prolonged MK2 activity inhibition, high kinase selectivity, and favorable in vivo anti‐tumor efficacy

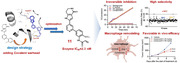

## 1 INTRODUCTION

Tumor‐promoting inflammation and immune evasion, recognized as cancer hallmarks, significantly fuel malignancy growth and progression.^1^ Inflammatory pathways, such as cGAS/STING, p38 mitogen‐activated protein kinase (p38‐MAPK), and JAK/STAT, play pivotal roles in carcinogenesis by modulating tumor immunity and the tumor immune microenvironment (TME). These pathways act as axes that may either drive or suppress tumor growth. In particular, p38 orchestrates the stress‐induced production of pro‐inflammatory cytokines, such as TNF‐α and interleukins, and stimulates enzymes like COX‐2.^2^, ^3^ Its dysregulation is implicated in a plethora of inflammatory diseases and tumors,^4^ triggering development of p38 inhibitors. Yet, their clinical utility is limited by severe systemic side effects—cardiac, hepatic, and central nervous system (CNS) toxicities—and by their limited efficacy.^5^, ^6^, ^7^, ^8^ Moreover, p38 kinase regulates numerous substrates integral to cellular homeostasis, leading to the uncontrollable outcomes of p38 inhibitors.^9^ Additionally, the efficacy of p38 inhibitors in treating inflammatory diseases, such as rheumatoid arthritis has been disappointing, partly due to tachyphylaxis^7^, ^8^, ^10^ that potentially arises from activated compensatory mechanisms when p38 is inhibited.^11^, ^12^ Consequently, attention has shifted to pivotal downstream proteins like mitogen‐activated protein kinase‐activated protein kinase 2 (MK2), which appear to be promising alternative therapeutic targets.

As the principal substrate of p38, MK2 largely governs the inflammatory pathological response by amplifying the production of pro‐inflammatory cytokines including IL‐1β, TNF‐α, IL‐6, and so on. Notably, MK2‐null mice do not experience the adverse health outcomes observed in p38α knockout mice.^13^ Significantly, MK2 inhibitors have shown effectiveness in preclinical models of ankylosing spondylitis, arthritis, and cancer, indicating potential therapeutic advantages over p38 inhibitors, which are further supported by sustained efficacy and fewer side effects in recent clinical findings.^14^, ^15^

The role of MK2 in promoting cancer is becoming increasingly recognized. As stated previously, MK2 is crucial in augmenting the production of pro‐tumorigenic inflammatory cytokines, TNF‐α, and IL‐6, particularly in macrophages, which play a central role in both initiating and resolving inflammation.^16^ Moreover, macrophages are prevalent within cancerous tissues, where an increased density of tumor‐associated macrophages (TAMs) is often indicative of a worse prognosis. TAMs typically exhibiting a phenotype similar to M2‐polarized macrophages, contributing to various aspects of carcinogenesis and tumor progression. They are implicated in promoting tumor cell proliferation and metastasis, inducing angiogenesis through vascular endothelial growth factor (VEGF) production, and fostering an immunosuppressive milie.^17^, ^18^, ^19^, ^20^ Previous study showed that, in the azoxymethane (AOM)/dextran sodiumsulfate (DSS)–induced colon cancer model, MK2 contributes to tumor progression by promoting an M2‐like protumorigenic and proangiogenic state in TAM.^21^ Furthermore, deletion of MK2 in dendritic cells has been shown to enhance CD8^+^ T‐cell response in melanoma model.^22^ All these indicate the important potential of MK2 in TME regulation as well as protumor‐associated inflammation. Additionally, sporadic reports show that high MK2 expression and activity correlate with poorer prognoses in patients with esophageal adenocarcinomas and head and neck squamous cell carcinomas.^23^, ^24^ Moreover, MK2 is implicated in resistance to anticancer therapies.^23^, ^25^, ^26^, ^27^

Given its significance, extensive efforts being devoted to developing MK2 inhibitors. Two types of MK2 inhibitors have been identified (see the representative MK2 inhibitors in Figure S1). ATI‐450, binding at the interface of p38α‐MK2 complex, is considered to be a selective MK2 inhibitor and showed significantly reduced potency against p38α alone. Studies have shown that ATI‐450 is over 700‐fold more potent for the p38α‐MK2 complex than for p38α‐PRAK or p38α‐ATF2.^28^ Another large class of MK2 inhibitors follows the traditional development pattern of kinase inhibitors by targeting the ATP site of MK2 kinase. However, these ATP‐competitive inhibitors showed limited MK2‐targeting potency and selectivity, which mainly attributes to the good affinity of ATP with MK2 and high concentration of ATP in cells.^29^, ^30^, ^31^, ^32^ This, at least in part, explains why numerous ATP‐competitive MK2 inhibitors, despite their impressive biochemical activity, did not advance into clinical trial. A practical strategy may lie in the development of covalent irreversible MK2 inhibitors, which would offer the advantages of potent, persistent, and highly selective inhibition. With this in mind, we initilized a campaign to develop a new series of irreversible inhibitors based on the unique residue Cys140 located at the hinge part in MK2. Following structure‐based design, synthesis, and optimization, we obtained a potent and selective irreversible MK2 inhibitor, compound **11**. It binds irreversibly to MK2 and exhibits nanomolar activity in inhibiting MK2 kinase activity with high selectiviy. It also demonstrated long‐lasting cellular MK2 signaling inhibitory efficacy and suppressed the production of MK2 classic downstream inflammatory cytokines. Moreover, compound **11** inhibits the M2‐like protumor phenotype of macrophages both in vitro and in vivo. And in the TAM‐dominated tumor models compound **11** treatment significantly delayed the tumor growth.

## 2 RESULTS

### 2.1 MK2 activity as a prognostic indicator

**FIGURE 1:**
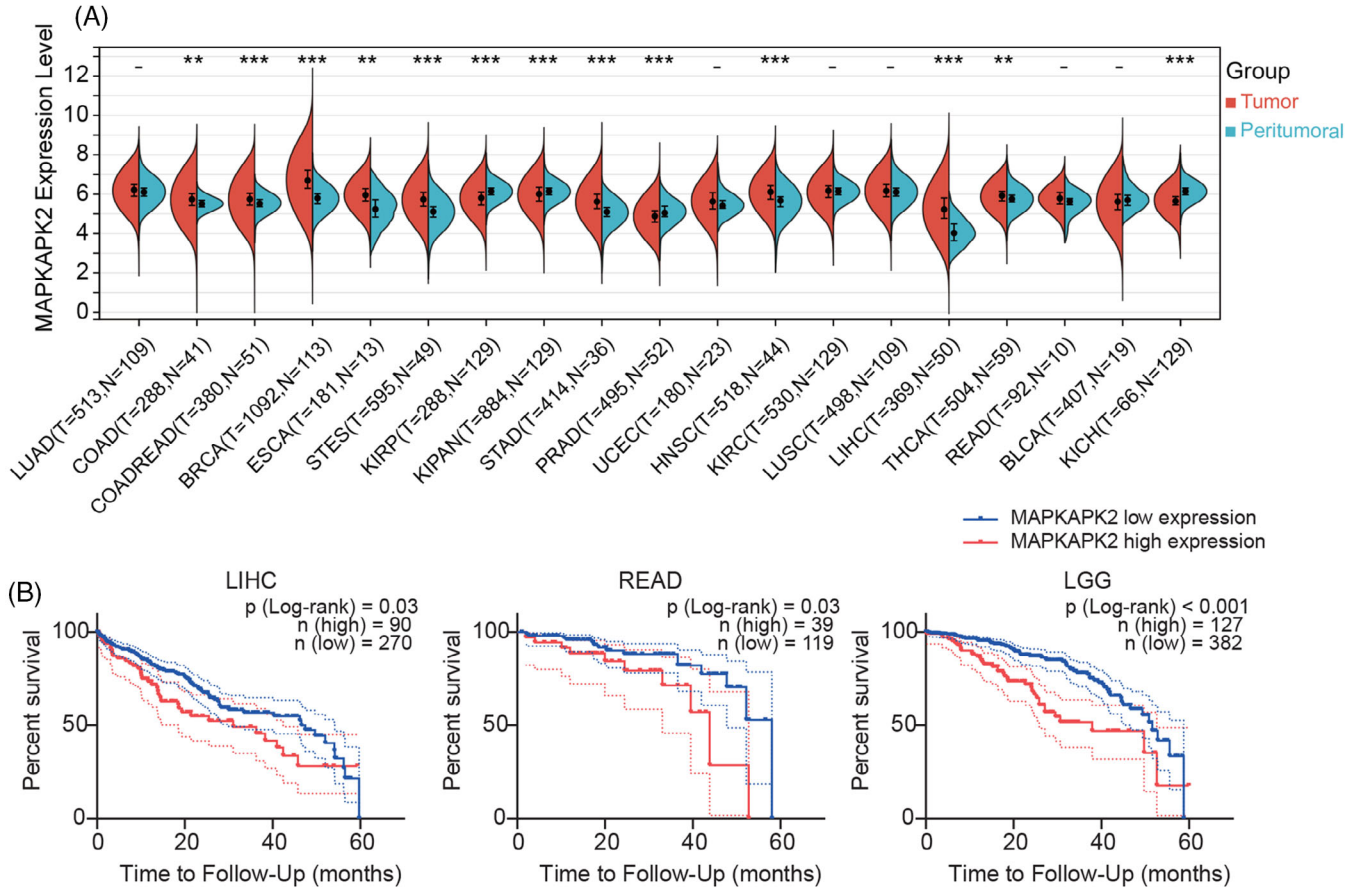
Association of mitogen‐activated protein kinase‐activated protein kinase 2 (MK2) activity with prognosis in cancer. (A) MK2 gene expression distribution in tumor versus adjacent peritumoral tissues across various cancer types, sourced from The Cancer Genome Atlas (TCGA) database. The *x*‐axis represents distinct cancer types, excluding samples with a size less than 10, while the *y*‐axis displays gene expression levels, with different colors indicating separate groups. Statistical differences between groups were evaluated using the Wilcoxon test, with significance levels marked as ^**^ *p* < 0.01, ^***^ *p* < 0.001. BLCA, bladder urothelial carcinoma; BRCA, breast invasive carcinoma; COAD, colon adenocarcinoma; COADREAD, colon adenocarcinoma/rectum adenocarcinoma; ESCA, esophageal carcinoma; HNSC, head and neck squamous cell carcinoma; KICH, kidney chromophobe; KIPAN, pan‐kidney cohort, KICH + KIRC + KIRP; KIRC, kidney renal clear cell carcinoma; LIHC, liver hepatocellular carcinoma; LUAD, lung adenocarcinoma; LUSC, lung squamous cell carcinoma; PRAD, prostate adenocarcinoma; READ, rectum adenocarcinoma; STAD, stomach adenocarcinoma; STES, stomach and esophageal carcinoma; THCA, thyroid carcinoma; UCEC, uterine corpus endometrial carcinoma. (B) Log‐rank survival analysis of patients with LIHC, READ, and LGG (lower grade glioma) patients according to the level of MK2 mRNA (high MK2: top 25%; low MK2: bottom 75%). Data sourced from TCGA database and the software GraphPad Prism 9.0 produced the figures.

Given the limited clinical reports on MK2 in tumors, we first explored the MK2 expression level across a spectrum of cancers using The Cancer Genome Atlas (TCGA) database. Notably, compared with peritumoral tissue, MK2 exhibits higher expression levels with statistical significance in 10 types of cancer, including colon adenocarcinoma, stomach and esophageal carcinoma, liver cancer and etc (Figure 1A), most of which are highly associated with inflammation and/or macrophage. Relative lower MK2 expression in tumor tissue with statistical significance was only observed in three types of kidney‐related cancer (KIRP, KIPAN, and KICH) and prostate cancer (PRAD). Additionally, within cancers commonly associated with inflammation, including liver hepatocellular carcinoma, rectum adenocarcinoma, and lower grade glioma (Figure 1B), a statistically significant correlation was found between high expression of MK2 and a patient's 5‐year survival, suggesting that high level of MK2 is associated with a bad prognosis. All these results robustly substantiate the critical role of MK2 in oncogenesis and underscore its potential as a promising therapeutic target, particularly in inflammation‐associated cancers.

### 2.2 Rational design and chemistry

**FIGURE 2:**
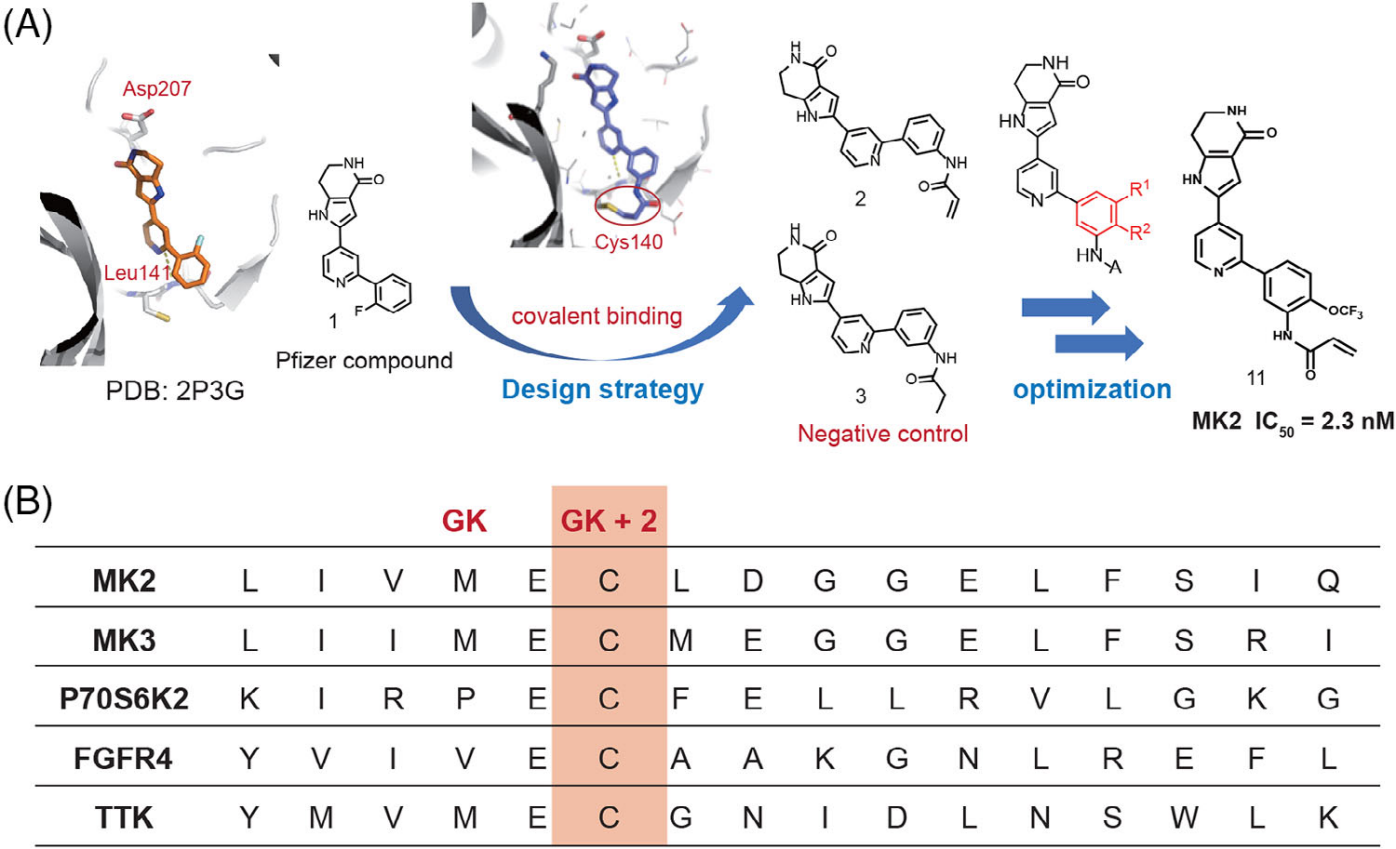
Rational design of novel MK2 inhibitors. (A) The design and optimization process. By analyzing the co‐crystal structure of compound **1** bound to MK2 (PDB: 2P3G), we utilized the docking method to design a covalent inhibitor (compound **2**), and compared with the negative control compound **3**. This series was optimized to identify the most potent inhibitor, compound **11**. (B) Five kinases having a cysteine located at two residues behind the gatekeeper.

Given the current challenges of ATP‐competitive MK2 inhibitors and the potential advantages of covalent inhibitors, we started with the analysis of the cocrystal structure of compound **1** ^33^ (developed by Pfizer) with MK2 protein (Figure 2A, left). At the MK2 ATP binding site, the pyridine ring of compound **1** forms an essential hydrogen bond with hinge residue Leu141. We scrutinized the residues surrounding the bound inhibitor, and identified a nearby cysteine residue Cys140 that could be utilized to design covalent binding inhibitors (Figure 2A, middle). Furthermore, we analyzed the sequences of kinase domains across whole human kinome and found that there were only five kinases including MK2 having a cysteine located at this position (two residues behind the gatekeeper (GK), Figure 2B), which implies that by forming the covalent bond with this cysteine we could also achieve high selectivity.

Based on above analysis, we thought this residue (Cys140) provided an opportunity to design selective covalent inhibitors for MK2, and the design and optimization processes are illustrated in Figure 2A. As shown, the N of the pyridyl formed an H‐bond with Leu141 and the distance between the phenyl ring of compound **1** and Cys140 is about 4.9 Å. Therefore, covalent inhibitor compound **2** was designed by in silico strategy consisting of protein modeling and molecular docking simulation. Docking study of compound **2** into X‐ray structure of MK2 showed that a hydrogen bond was formed between the pyridine and Leu141 near the hinge binding area. Moreover, the defined covalent bond between the Michael acceptor covalent group and Cys140 near the hinge binding area does not disturb the other interactions (Figure S2A). Thus, we synthesized compound **2** to validate our design. Also, a negative control compound **3** was synthesized using a propionamide group to replace acrylamide for comparison (Figure 2A).

Through enzymatic assessment (Table S1) we found that compound **2** containing an acrylamide group at the *m*‐position of the phenyl ring showed the activity with IC_50_ value of 728.1 nM in the MK2 enzymatic assay. But the negative control compound **3** showed much weaker activity with only about 30% inhibition at 1 µM concentration, which implied compound **2** forms the covalent bond between the acrylamide group and the cysteine residue at hinge part. To improve the inhibition activity, modifications were made on the phenyl ring of compound **2** to enhance the covalent binding (as shown in Figure 2A). Generally, the compounds with substitutions at R^2^ (**5**, **9**, **11**, and **13** in Table S1) exhibited stronger inhibition than those at R^1^ (**4**, **8**, **10**, and **12** in Table S1), except the compounds **6** and **7** with methyl group. Electron‐withdrawing groups such as trifluoromethyl and trifluoromethoxy and electron‐donating groups such as methoxyl, methyl, and dimethylamino group were found no significant differences in enzymatic activities. Among all these compounds **11** with trifluoromethoxy at R^2^ showed particularly strong inhibition with IC_50_ = 2.3 nM, which is more potent than the well‐studied MK2 inhibitors PF‐3644022 and CC‐99677. Thus, adopting the rational design approach and building upon the preliminary reversible inhibitor **1**, we successfully discovered a series of covalent MK2 inhibitors. Among these, compound **11** stands out as the most potent inhibitor and was chosen for further characterization. Additionally, the docking study demonstrated that the binding interactions of compound **11** closely mirrored those observed in compound **2** (Figure S2B).

### 2.3 Compound **11** is an irreversible and selective MK2 inhibitor

**FIGURE 3:**
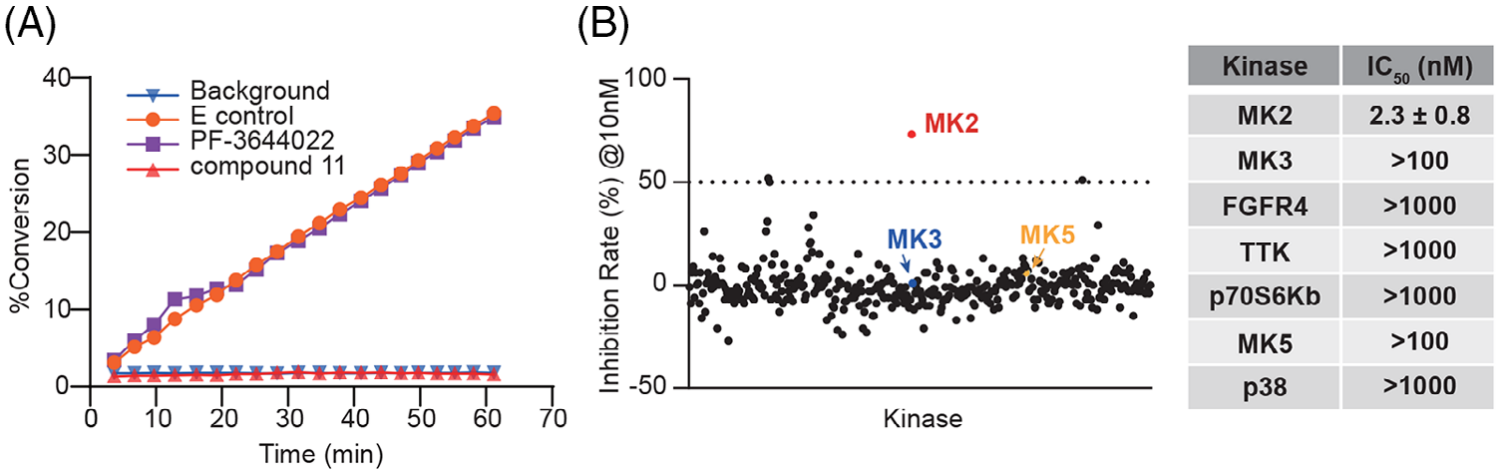
High selectivity and irreversible inhibition on MK2 by compound **11**. (A) Compound **11** inhibits MK2 kinase activity irreversibly. The phosphorylation rate of the substrate peptide, expressed as the conversion rate, illustrates the extent of inhibition. The displayed results reflect the phosphorylation outcomes of the peptide by MK2 kinase following preincubation with the specified inhibitor. The group preincubated without MK2 kinase as background group, or preincubated only with MK2 kinase without inhibitor as E control group. (B) An extensive kinase selectivity profile for compound **11**, highlighting its specificity for MK2 against 380 other human recombinant kinases.

To assess its role as an irreversible MK2 kinase inhibitor, we conducted a kinetic study on compound **11**. The well‐known reversible ATP‐competitive MK2 inhibitor, PF‐3644022, served as a negative control. In this test, MK2 kinase was initially incubated with either compound **11** or PF‐3644022 for 30 min at a final concentration equivalent to 100 times the IC_50_ value. The kinase‐inhibitor mixture was subsequently diluted by a factor of 100 with a reaction buffer that included both substrate peptide and ATP. Under these circumstances, the activity of MK2 kinase was continuously monitored by assessing the phosphorylated substrate percentage. Although preincubated with PF‐3644022, the kinase activity of MK2 recovered gradually (Figure 3A), as the kinase activity curve is substantially overlapped with that of the E control group, in which the MK2 kinase was only pre‐incubated with the vehicle buffer. However, preincubation with compound **11** resulted in sustained and potent inhibition of kinase activity of MK2 (Figure 3A). The enzyme activity curve almost overlapped with the curve of background group, which included only the substrate peptide without inhibitors and MK2 kinase. This suggests that compound **11** has the potential to irreversibly bind to MK2, thereby inhibiting its kinase activity.

Subsequently, to ascertain whether the high potency of compound **11** was specific for MK2, an additional 380 human recombinant protein kinases were screened. At a concentration of 10 nM, compound **11** exhibited negligible inhibitory effects on the other 380 kinases tested, all with inhibitory rate less than 50% (Figure 3B). Additionally, in contrast to its high potency against MK2 (IC_50_ = 2.3 ± 0.8 nM), compound **11** exhibited more than 434‐fold (91.8% of total) or 43‐to‐434‐fold (5.8%) selectivity over the other tested kinases (Table S2). Among them, compound **11** showed no obvious inhibition on p38 kinase even at 1 µM concentration (inhibition rate was less than 10%) (Table S2). Additionally, for the above‐mentioned four kinases (family member MK3, p70S6Kb, FGFR4, and TTK), which share a sequence homologous cysteine to C140 of MK2 and another MAPKAPK family member MK5, compound **11** also showed no obvious inhibitory effect even at the highest tested concentration (Figure 3B, right panel and Table S2). These results collectively underscore compound **11**’s remarkable selectivity for MK2, highlighting its specificity in targeting MK2 kinase.

### 2.4 Compound **11** profoundly suppressed cytokine transcription and production in macrophages, targeting classic downstream genes of MK2

**FIGURE 4:**
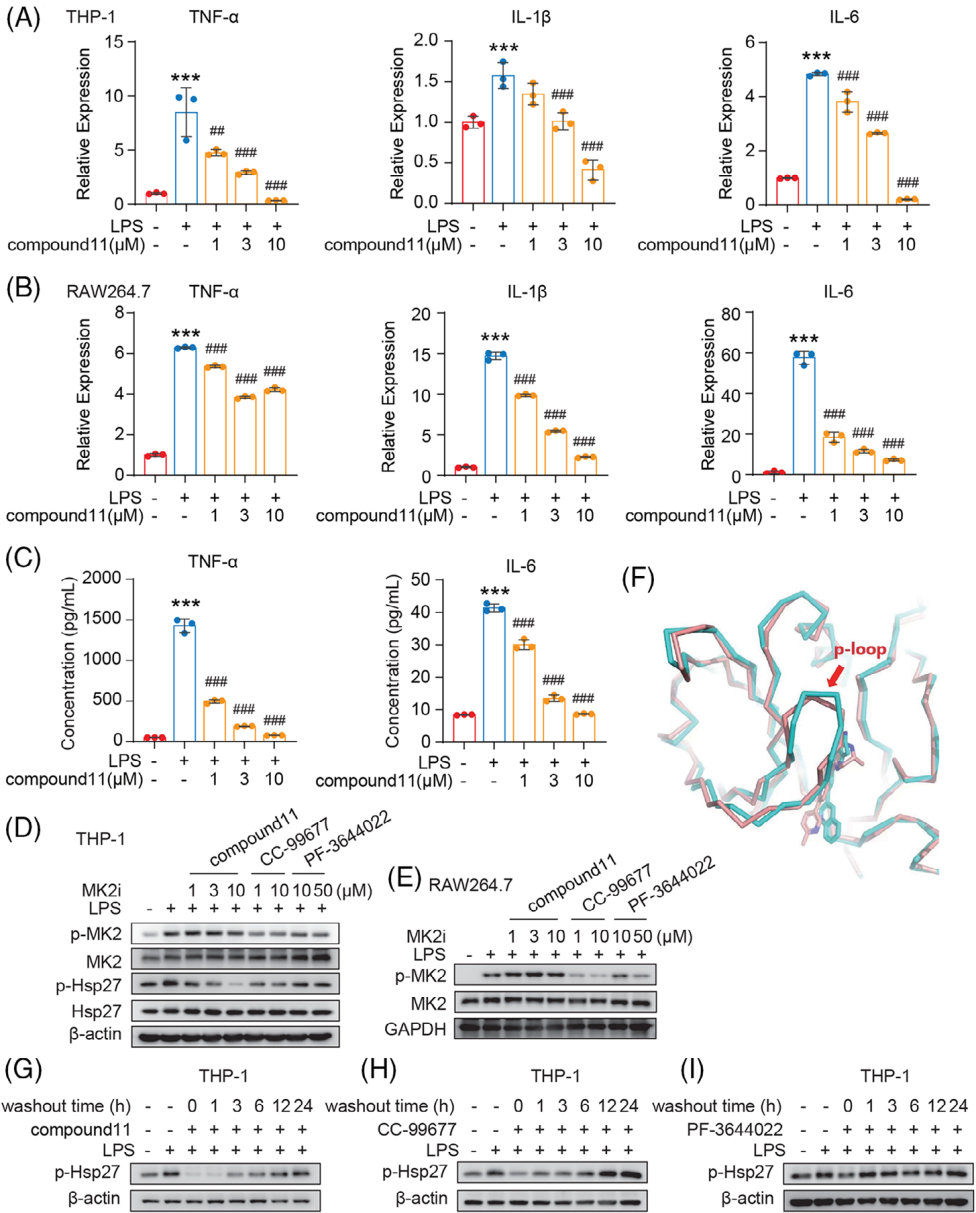
Targeting cellular MK2 signaling with irreversible inhibition by compound **11**. (A and B) RT‐PCR analysis of IL‐1β, TNF‐α, and IL‐6 expression in Lipopolysaccharides (LPS)‐stimulated THP‐1 (A) and RAW264.7 (B) Cells, with pre‐treatment of compound **11** for 3 h followed by LPS (3 h) stimulation. (C) The inhibitory effects of compound **11** on TNF‐α and IL‐6 production in LPS‐stimulated RAW264.7 macrophages for 24 h, measured by ELISA. (D and E) Compound **11**, alongside CC‐99677 and PF‐3644022, suppressed MK2 pathway activation, validated through western blot analysis post‐LPS stimulation. Cells were preincubated with the specified compounds for 1 h prior to LPS stimulation (100 ng/mL, 30 min). (F) Distinguishes the mode of action between compound **11** and CC‐99677. The complex structure of a small molecule and MK2 (3R2Y) with the same parent nucleus as compound **11** is shown in cyans, and the complex structure of a small molecule and MK2 (3FYJ) with the same parent nucleus as CC‐99677 is shown in orange. (G–I) The effect of indicated inhibitor on Hsp27 phosphorylation following inhibitor washout. Cells were pretreated with indicated compounds for 1 h and then washed to remove free compound and incubated for a further indicated time before activation with LPS. In (A–C), LPS (100 ng/mL) served as the stimulation control, while untreated cells were the baseline control. The data represent as mean ± SD from triplicates, with statistical analysis conducted via one‐way analysis of variance (ANOVA) (^##^ *p* < 0.01, ^###^ *p* < 0.001 vs. stimulation control; ^***^p < 0.001 vs. baseline control).

MK2 is recognized for inducing the production of pathological cytokines that fuel inflammation, including IL‐6, TNF‐α, and IL‐1β. These cytokines are the classic downstream target gene of MK2 and are identified as significant contributors to the risk of inflammation‐driven cancer,^34^, ^35^, ^36^ such as colorectal cancer and hepatocellular carcinoma. Therefore, we assessed the transcriptional levels of these typical downstream cytokines in human monocytic THP‐1 cell‐line‐derived macrophage and the mouse RAW264.7 macrophage cell line under the influence of compound **11**. As expected, Lipopolysaccharides (LPS) significantly increased the expression of TNF‐α, IL‐6, and IL‐1 mRNA, and such increase was significantly inhibited by compound **11** in a concentration‐dependent manner across both cell types (Figure 4A,B). Correspondingly, the secretion level of IL‐6 and TNF‐α by LPS‐stimulated RAW264.7 cells were similarly reduced by compound **11** (Figure 4C). Taken together, our findings corroborate that compound **11** effectively impedes the transcription and production of these key MK2 downstream pro‐inflammatory cytokines.

Next, the efficacy of compound **11** in cellular MK2 signaling inhibition was evaluated by examining both p‐MK2 and p‐Hsp27 at Ser82 levels, the latter being a direct downstream substrate of p‐MK2. As shown in Figure 4D, in LPS‐stimulated THP‐1 derived macrophages, compound **11** significantly inhibited the phosphorylation of Hsp27. The activity of compound **11** was much more potent than that of PF‐3644022 and CC‐99677. Interestingly, it was found that compound **11** did not inhibit the MK2 phosphorylation at T334 site phosphorylated by p38, which was also inhibited by the treatment with PF‐3644022 or CC‐99677. Similar result was observed in the mouse RAW264.7 macrophages (Figure 4E). We try to explore this interesting phenomenon by superimposing the complex crystal structure (3FYJ) that harboring the same skeleton as CC‐99677 (Figure 4F). It can be found that the overall matching of the protein is good with the backbone root‐mean‐square deviation (RMS) value only 0.78 Å, but further comparing the ATP binding site, the P‐loop structure of 3FYJ is closer to the inhibitor. The reason may be that CC‐99677 possesses a larger and more rigid framework compared to compound **11**, impacting the outward movement of the N‐terminal β‐sheet structure. In that way, CC‐99677 binding to MK2 kinase after pre‐incubation with cells and after LPS stimulation may cause the difference in the spatial conformation of MK2 kinase domain, which may make p38 cannot properly bind to MK2, resulting in a decrease in the phosphorylation of MK2. However, compound **11** has stronger covalent properties and a more flexible backbone, so its binding to MK2 does not affect the phosphorylation of MK2 by p38.

Furthermore, to validate the irreversible mechanism of action of compound **11** at the cellular level, we performed a cellular washout assay and assessed p‐Hsp27 levels. The established MK2 irreversible inhibitor CC‐99677 served as positive control, while the reversible ATP‐competitive inhibitor PF‐3644022 was used as negative control. We found that 3 h after compound **11** was washed out, p‐Hsp27 level was still significantly inhibited though its level began to recover slightly. And till 24 h after compound **11** washout, the p‐Hsp27 recovered significantly (Figure 4G). Similarly, the level of p‐Hsp27 was significantly inhibited and only almost recovered until 12 h after CC‐99677 withdrawal (Figure 4H). In contrast, only 1 h after PF‐3644022 washout, p‐Hsp27 level was totally recovered (Figure 4I). Post washout, both compound **11** and CC‐99677, but not PF‐3644022, maintained their capacity to inhibit Hsp27 phosphorylation, indicating that compound **11** binds irreversibly to MK2 within cells. Additionally, compound **11** demonstrated a more sustained effect than CC‐99677.

### 2.5 Both compound **11** and MK2 knockdown effectively suppressed the protumor M2‐like polarization of macrophages in vitro and in vivo

**FIGURE 5:**
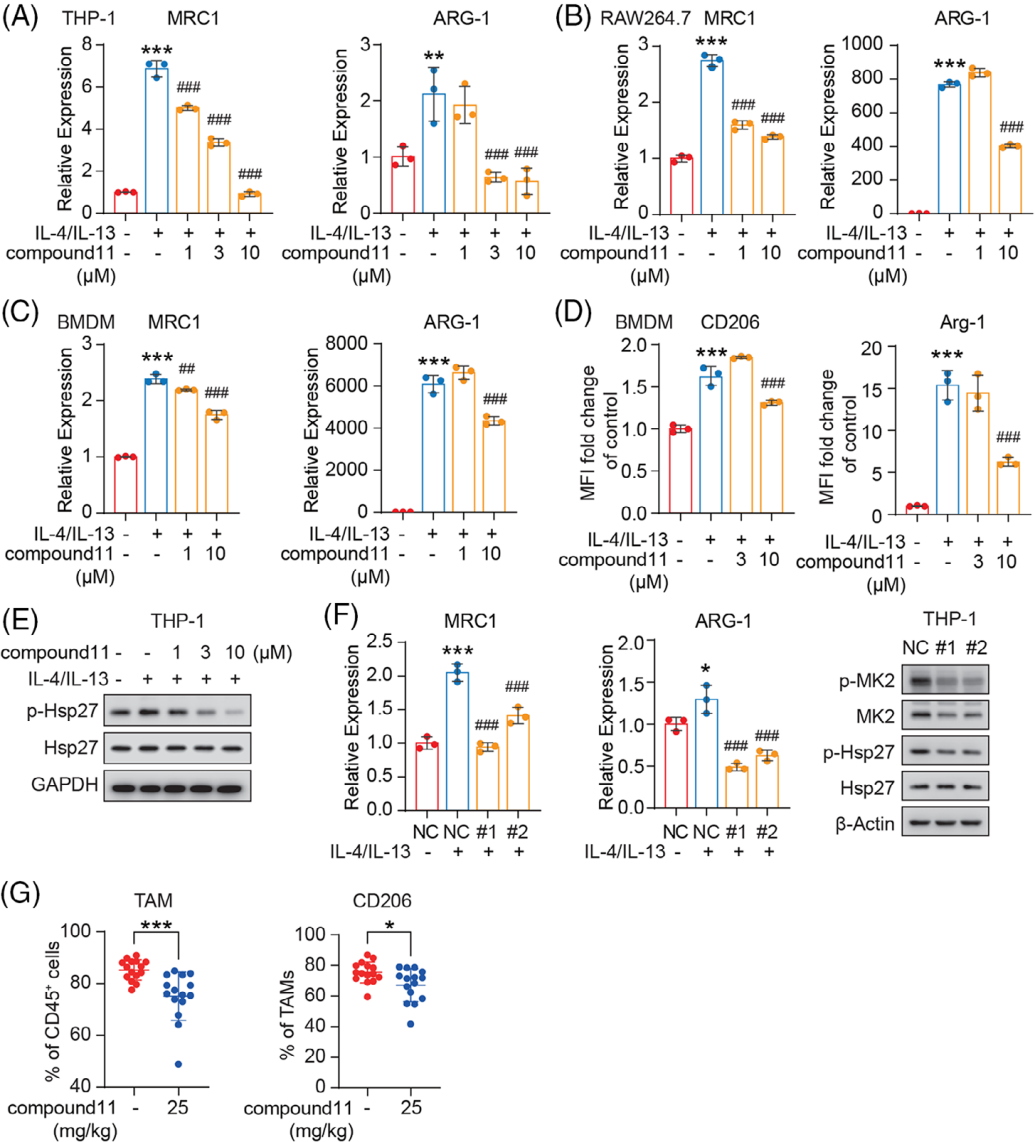
Inhibition of pro‐tumorigenic M2‐like polarization in macrophages by compound **11**. (A–C) RT‐PCR analysis highlights the impact of compound **11** on MRC1 and ARG‐1 mRNA expression in various macrophage cells treated with IL‐4/IL‐13 alone or combined with compound **11** for 12 h or 24 h. (D) CD206 and Arg‐1 expression in bone marrow‐derived macrophages (BMDM) cells via flow cytometry post 48 h treatment of compound **11** and IL‐4/IL‐13. (E) The treatment effects of compound **11** in THP‐1 cells using western blot analysis. Cells were pretreated with compound **11** for 1 h and followed by IL‐4/IL‐13 treatment (30 min). (F) The influence of MK2 siRNA on MRC1 and ARG‐1 mRNA expression in THP‐1 cells by RT‐PCR analysis post‐IL‐4/IL‐13 stimulation (12 h). (G) Flow cytometric analysis of tumor infiltrating tumor‐associated macrophages (TAMs) and CD206^+^ TAMs in the MC38 tumor model treated with vehicle or compound **11** for 9 days (*n* = 15 mice per group). Data are shown as the mean ± SD. ^*^ *p* < 0.05; ^***^ *p* < 0.001 versus the vehicle group, determined by unpaired *t* test. In A–D and F, IL‐4/IL‐13 (20 ng/mL) served as the stimulation control, while untreated cells were the baseline control. Data represent as mean ± SD from triplicates, with statistical analysis conducted via one‐way analysis of variance (ANOVA) (^##^ *p* < 0.01, ^###^ *p* < 0.001 vs. stimulation control; ^*^ *p* < 0.05 ^***^ *p* < 0.001 vs. baseline control).

A previous study has reported that MK2 fostered M2‐like polarization of TAMs, driving a protumor and proangiogenic phenotype which in turn accelerates tumor progression.^21^ We thus assessed the influence of compound **11** on macrophage polarization and function. Our experiments used human THP‐1 monocyte induced macrophages, mouse RAW264.7 macrophages, and mouse bone marrow‐derived macrophages (BMDM). These cells were induced into M2‐like macrophages using the classic IL‐4 and IL‐13 stimuli. As anticipated, the efficiency of M2 polarization was markedly enhanced by IL‐4 and IL‐13 (Figure 5A–C), as indicated by a substantial increase in mRNA expression levels of MRC1 (encoding CD206) and ARG‐1 (encoding Arginase‐1, Arg‐1), both of which are typical M2 markers. Notably, these conditions yielded elevated levels of p‐MK2 signaling (Figure S3A). Moreover, with the introduction of MK2 inhibitor compound **11**, the transcriptional level of both MRC1 and ARG‐1 were significantly suppressed (Figure 5A–C). Further evaluation via flow cytometry indicated that compound **11** could also suppress the expression levels of surface CD206 and Arg‐1 in representative BMDM stimulated with IL‐4/IL‐13 (Figure 5D). A similar result was noted in IL‐4/IL‐13 stimulated RAW264.7 cells assessed by flow cytometry (Figure S3B). In the case of representative THP‐1 cells, as we expected, alongside the suppression of M2 polarization in macrophages by compound **11**, there was a significant inhibition of p‐Hsp27 within the macrophages (Figure 5E).

Following this line of investigation, we employed siRNA for the transient knockdown of MK2 in THP‐1 monocyte‐ differentiated macrophages. Similar to the effects observed with compound **11** treatment, MK2 signaling inhibition was also associated with a marked reduction in MRC1 and ARG‐1 mRNA levels in IL‐4/IL‐13 stimulated macrophages where MK2 was knocked down (Figure 5F). M2 macrophages play a pivotal role in promoting tumor angiogenesis through the secretion of critical factors like VEGF, which facilitate tumor neovascularization.^17^ As shown in Figure S3C, compound **11** inhibited the relative mRNA level of VEGF in a dose‐dependent pattern in the IL4/IL13‐stimulated macrophages. These findings further validate the significance of MK2 in the M2 polarization of macrophages and illustrate how compound **11**, by targeting MK2, suppresses the pro‐tumorigenic phenotype of macrophages.

Next, we explored the effect of compound **11** on the M2 polarization of TAM in vivo. Mice bearing TAM‐dominant murine MC38 colorectal tumor model were treated with compound **11**, followed by an analysis of TAM infiltration within tumor tissues. Treatment with compound **11** resulted in a marked reduction in both the total and “protumor” M2‐like TAM populations, as evidenced by the decreased surface expression of CD206. This finding is in alignment with our in vitro observations (Figure 5G).

Taken together, these findings imply that compound **11** is capable of reversing the tumor‐promoting M2‐like polarization of macrophages.

### 2.6 Compound **11** slows tumor growth in the MC38 tumor model

**FIGURE 6:**
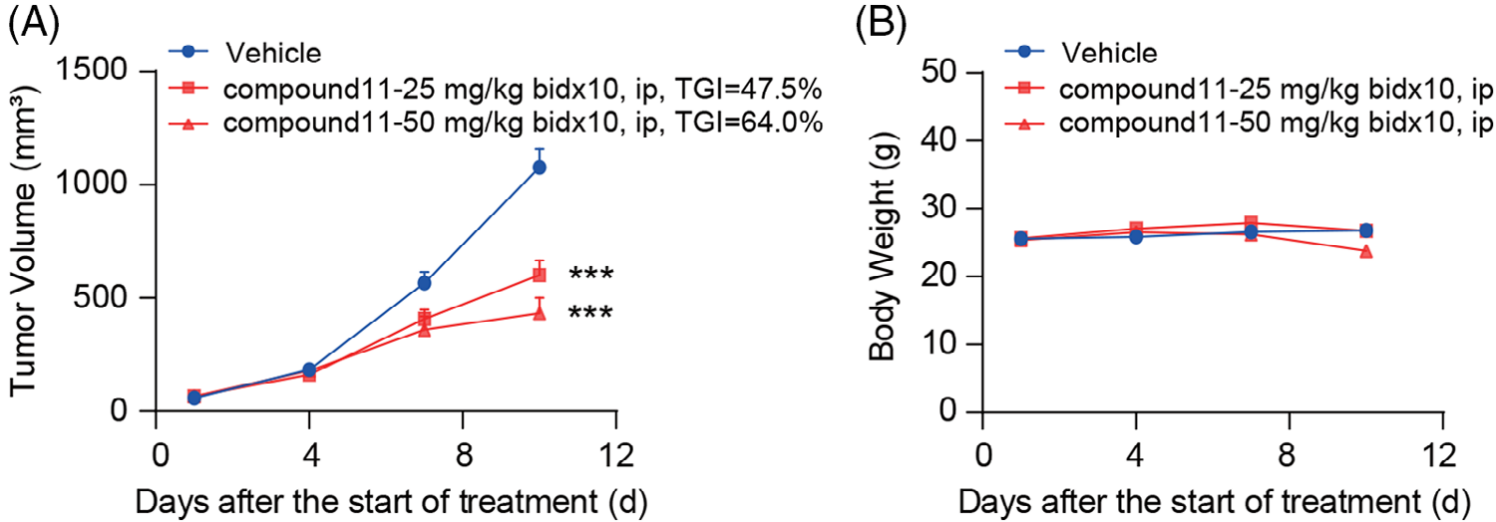
Delayed tumor growth in the MC38 model by compound **11**. Over a course of 10 days, immune‐competent mice bearing MC38 tumors were treated intraperitoneally with either compound **11** or a vehicle control (*n* = 7/group). The tumor growth trend (Panel A) and the body weight data (Panel B) are displayed as the mean ± SEM. Statistical analysis through one‐way analysis of variance (ANOVA) revealed significance as ^***^ *p* < 0.001.

Further, we evaluated the in vivo antitumor efficacy of compound **11** in MC38 tumor model, utilizing immune‐competent mice. Preliminary pharmacokinetic assessment was conducted on mice; following an intraperitoneal injection of 10 mg/kg of compound **11**, the peak concentration (*C*_max_) and half‐life (*T*_1/2_) were determined to be 125.87 ng/mL and 0.38 h (Table S3), respectively, indicating rapid metabolism in mice. Consequently, the regimen for in vivo administration was adjusted to twice daily. MC38 tumor‐bearing mice were then intraperitoneally administrated with either compound **11** or vehicle control, also twice daily, tumor volume, and body weight were measured (Figure 6A,B). We found that compound **11** significantly hindered tumor growth. Specifically, the rates of tumor growth inhibition rates at dosages of 25 and 50 mg/kg compound **11** were 47.5% and 64.0%, respectively, both achieving statistical significance (Figure 6A).

## 3 DISCUSSION

MK2, a crucial downstream substrate of p38, regulates the inflammatory pathological response predominantly triggered by p38. It is expected to circumvent the serious adverse effects typically correlated with p38 inhibition, while displaying sustained efficacy. Thus, it has emerged as a promising alternative drug target to p38.

Currently, various reported MK2 inhibitors are targeting the ATP binding site, and typically featuring a nitrogen‐containing ring motif capable of forming hydrogen bonds with the hinge part of the MK2 kinase domain. However, ATP‐competitive MK2 inhibitors, despite their excellent biochemical activity, have largely failed to progress into clinical trials. This is partly because the ATP binding affinity to MK2 is approximately 30 µM,^31^, ^32^ and cellular ATP concentrations range from 0.5 to 5 mM,^29^, ^30^ suggesting that even the nanomolar MK2 inhibitors are difficult to compete with the intrinsic ATP molecules. Recently, covalent kinase inhibitors, such as EGFR inhibitor osimertinib and BTK inhibitor ibrutinib, exhibit good clinical benefits over the reversible ATP comeptitive inhibitors, largely because they can block the enzymatic activity for a long time and achieve good selectivity by bonding with the position‐specific cysteine residue.

Therefore, we intended to develop irreversible inhibitors capable of persistently occupying the ATP‐binding site to achieve the desired inhibition effect. Through analysis of cocrystal structure and covalent docking study, we found that the designed prototype compound **2** indeed showed good inhibition activity. Subsequent modifications to the phenyl ring of compound **2** led to enhanced covalent binding, which culminated in the development of compound **11** with nanomolar potency against MK2 kinase.

Docking study confirmed that the binding interactions of compound **11** were very similar to those of compound **2**. Biochemical and cellular assays further verified that compound **11** irreversibly inhibited MK2, exhibiting prolonged efficacy characteristic of a covalent inhibitor. The irreversible covalent action mechanism of such series compounds ensures high selectivity. In contrast to its high potency against MK2 kinase (IC_50_ = 2.3 ± 0.8 nM), compound **11** demonstrated over 434‐fold (91.8% of total) or 43‐ to 434‐fold (5.8%) selectivity for MK2 over the other tested 380 human protein kinase. Notably, it exhibited more than 434‐fold selectivity for MK2 over p38. Thus, as a selective MK2 inhibitor, compound **11** has the potential to optimize therapeutic targeting of MK2 while avoiding the severe toxic effects associated with p38 targeting in patients. The high selectivity potentiates these MK2 inhibitors as promising candidates for combination therapies.

MK2, as a pivotal regulator of the well‐acknowledged p38 inflammation signaling pathway, is increasingly recognized for its critical role in the initiation and progression of cancer. Through an analysis of the clinical data from the TCGA database, we found the significant impact of MK2 on cancer. It was revealed that MK2 is highly expressed in a variety of cancers, especially those closely linked to inflammation. Notably, in cancers commonly associated with inflammation, a high level of MK2 expression within tumors significantly correlates with poorer prognosis (Figure 1B). However, investigation into the MK2 gene dependency across 1024 different tumor cell lines in the DepMap database highlighted that the survival of the majority of these tumor cell lines does not depend on MK2 per se (Figure S4). In line with this, compound **11** displayed minimal cytotoxic effects, with IC_50_ values ranging from approximately 30–50 µM in cytotoxicity assays involving three normal cell lines and six tumor cell lines, including MC38 (Table S4), further confirming a limited direct impact of MK2 inhibition on cytotoxicity. Notably, MK2 is highly expressed in macrophages that play a vital role in both activation and resolution of inflammation.^37^ Moreover, macrophages are abundant in cancer, and such abundance correlates with poor prognosis in most malignancie.^17^, ^18^, ^19^, ^20^ In this study, we noted an elevated p‐MK2 level in M2‐like macrophages, which typically exhibit a protumor phenotype. Treatment with compound **11** consistently suppressed this phenotype of macrophage, as evidenced by downregulated classic M2 marker Arg‐1 and CD206 as well as the key pro‐angiogenic factor VEGF. Additionally, the knockdown of MK2 using specific siRNA in macrophages also led to the downregulation of M2 markers. In vivo studies further revealed a significant reduction in tumor‐infiltrating M2‐like macrophages within the TAM‐rich murine MC38 tumor model following treatment with compound **11**. Moreover, compound **11**’s effect on macrophages extended to inhibition of typical proinflammatory cytokines and crucial factors in inflammation‐driven tumorigenesis. These observations suggest that the primary mechanism through which compound **11** inhibits the growth of MC38 xenografts is likely through modulating TAM activity, rather than direct cytotoxic effects on tumor cells. These results, aligned with findings from sporadic studies,^21^, ^38^ collectively underscore the crucial role of MK2 in macrophage biology, especially in promoting an M2‐like phenotype that supports tumor growth and angiogenesis, as well as enhancing tumor‐promoting inflammation. These findings bolster the rationale for pursuing the development and application of MK2 inhibitors in the treatment of inflammation‐driven or macrophage‐rich cancers.

Nonetheless, compound **11** still had some limitations for further development. It demonstrated poor pharmacokinetic properties, as evidenced by a short half‐life in vivo, and necessitating intraperitoneal administration. Based on these findings, the next step involves enhancing the metabolic properties of this series of compounds post‐oral administration, aiming to discover MK2 inhibitor candidates with improved pharmacokinetic profiles and elevated activity.

## 4 MATERIALS AND METHODS

### 4.1 Cell culture

The THP‐1, RAW264.7, and MC38 cell lines, sourced from the American Type Culture Collection (ATCC), were acquired between 2000 and 2017. All cell lines were cultured following the provided guidelines. The human‐derived cell lines were authenticated employing short tandem repeat markers by Genesky Biopharma Technology. While for the mouse‐derived cell lines, single nucleotide polymorphism was employed for characterization by Crown Bioscience, with the most recent verification conducted in 2023.

### 4.2 Kinase inhibition assay

To assess the impact of various compounds on MK2 kinase activity, we utilized the ADP‐Glo Assay Kit (Promega) following the manufacturer's instructions. Luminescence measurements were taken using a SpectraMax Paradigm reader. The inhibition percentage was calculated using the following formula: ((specific signal_DMSO control_ − specific _signal inhibitor_)/(specific signal_DMSO control_)) × 100%. The IC_50_ values were determined employing dose–response curve fitting via Prism (Graph Pad). Additionally, Eurofins company (Celle Lévescault) conducted the analysis on compound **11**’s inhibitory activity against 380 other human recombinant kinases at various concentrations.

In the kinase kinetic analysis to elucidate the irreversible inhibition of MK2 by compound **11**, a Mobility Shift Assay was conducted. In this approach, after pre‐incubating MK2 kinase (2 nM) with an excess of compound **11**, CC‐99677, or PF‐3644022 for 30 minutes at room temperature at a concentration 100 times the IC_50_, the mixture was diluted to a 1× reaction solution containing the substrate peptide and ATP (262 µM). The EZ Reader (Caliper Life Sciences, MA) was then used to assess the kinase activity of the mixture, which was monitored continuously for 1 hour.

### 4.3 Western blot analysis

After the treatment outlined in the figure legends, cells were lysed using 1× sodium dodecyl sulfate (SDS) solution. The lysates were then subjected to SDS‐polyacrylamide gel electrophoresis for separation, followed by transferring onto nitrocellulose membranes. The membranes were incubated with primary antibodies specific to phospho‐MK2, phospho‐Hsp27, MK2, Hsp27, GAPDH, and β‐actin (Cell Signaling Technology), followed by horseradish peroxidase‐conjugated anti‐mouse or anti‐rabbit IgG secondary antibodies. Protein bands were detected using an enhanced chemiluminescence detection reagent (Thermo Fisher Scientific).

### 4.4 Quantitative real‐time PCR (RT‐PCR) analysis

Total RNA was extracted from cultured cells using the EZ‐press RNA Purification Kit (EZBioscience). Complementary DNA was synthesized using the ABScript III RT Master Mix for qPCR with gDNA remover (ABclonal Technology) and was quantified with primers listed in Table S5. The relative mRNA expression levels were quantified through qPCR using the SYBR green gene expression assay (ABclonal Technology).

### 4.5 siRNA transfection

Transfections were carried out using Lipofectamine RNAiMAX reagent (Invitrogen) following the guidelines provided by the manufacturer. The double‐stranded nucleotide sequences utilized in siRNA transfection were specifically designed to induce MK2 knockdown. The sequence of the siRNA used is detailed in Table S6.

### 4.6 Cytokine production by ELISA

Following collection and centrifugation of the cell culture media, supernatants were harvested and the levels of cytokine secretion were evaluated using specific ELISA kits (absin), following the manufacturer's protocol.

### 4.7 Analysis of macrophages by flow cytometry

In the in vitro analysis, cells were harvested, stained with surface antibodies, and subsequently fixed and permeabilized using IC Buffer following manufacturer's guidelines. Antibodies targeting intracellular molecules were used for further staining as cells were incubated for an additional 30 min at 4°C. These cells were then filtered using a 300‐mesh cell sieve prior to analysis via CytoFLEX (Beckman Coulter Life Sciences).

The study of tumor‐infiltrating macrophages involved the fragmentation and digestion of MC38 mouse tumors using a Mouse Tumor Dissociation Kit (Miltenyi). Post‐digestion, the cells were filtered through a 70‐µm cell strainer and subsequently stained with fluorescent‐labeled antibodies, replicating the aforementioned in vitro procedure. The prepared samples were then tested using a BD LSRFortessa.

Antibodies targeting specific proteins along with corresponding isotype controls or FMO controls were employed to examine the leukocyte infiltrate: CD11b, CD45, CD206, Arg‐1, and F4/80 (BD, eBioscience and Biolegend). A LIVE/DEAD Fixable Violet Dead Cell Stain Kit (Invitrogen) was used to assess cell viability. Eventually, the gathered data were analyzed using the FlowJo software, with the gating strategies utilized for flow cytometry represented in Figure S5.

### 4.8 Mouse pharmacokinetics study

Compound **11** was dissolved in DMSO/Tween 80/PEG400/NaCl (5/5/45/45, v/v/v/v) to a concentration of 5 mg/mL and given to ICR mice (male, 18−22 g, *n* = 3) by intraperitoneal injection. Blood samples were obtained at intervals of 0.25, 0.5, 1, 2, 4, and 8 h following administration. The sample preparation and the analysis were following the protocol provided in the Supporting Information Methods.

### 4.9 In vivo antitumor evaluation

C57BL/6 mice, aged 6 weeks, were house and cared for in a controlled, specific pathogen‐free environment. MC38 tumor cells (3.5 × 10^5^ in 100 µL) were administered to the mice via subcutaneous injection into the right flanks. Upon reaching a tumor volume between 50 and 100 mm^3^, mice were randomized and assigned to either the vehicle control group or the specified treatment group (*n* = 7/group). The details regarding the administration of inhibitors and the measurement of tumors are delineated in the Supporting Information Methods.

### 4.10 Statistical analyses

Statistical analyses were carried out using GraphPad Prism 9.0 software, utilizing either a *t*‐test or one‐way analysis of variance with Tukey's test, Dunnett's multiple‐comparison test, or the Wilcoxon test as appropriate.

## AUTHOR CONTRIBUTIONS

Jing Ai and Bing Xiong conceived and designed this project. Jing Ai and Bing Xiong designed experiments and supervised data analysis. Danyi Wang, Deqiao Sun, Danqi Chen, Bing Xiong, and Jing Ai analyzed the data and wrote the paper. Danyi Wang, Deqiao Sun, and Danqi Chen drew figures and tables. Bing Xiong conducted molecular docking and rational design. Xiaoyan Wang, Lu Tang, Qichang He, and Danqi Chen synthesized the target compounds and key intermediates. Danyi Wang, Deqiao Sun, Yinchun Ji, Xia Peng, Ye Yang, and Xuan Zhou performed in vitro and in vivo experiments of MK2 inhibition and antitumor efficacy. All authors have given approval to the final version of the manuscript.

## CONFLICT OF INTEREST STATEMENT

The authors declare no conflicts of interest.

## ETHICS STATEMENT

Animal procedures were approved by the Institutional Animal Care and Use Committee of the Shanghai Institute of Materia Medica (Nos. 2022‐10‐GMY‐31 and 2022‐01‐YY‐21).

## Supporting information

Supporting Information

## Data Availability

The authors confirm that the data supporting the findings of this study are available within the article and its Supporting Information Materials.
