# Supplementary material for: Remodeling tumor‐associated macrophage for anti‐cancer effects by rational design of irreversible inhibition of mitogen‐activated protein kinase‐activated protein kinase 2
Source: MedComm (2020). 2024 Jul 10;5(7):e634. doi: 10.1002/mco2.634 (PMC11233931; doi:10.1002/mco2.634)

**Supplemental information**

**Remodeling Tumor-associated Macrophage for Anti-cancer Effects by Rational Design of Irreversible Inhibition of Mitogen-Activated Protein Kinase-Activated Protein Kinase 2**

Danyi Wang^1,4,#^, Deqiao Sun^1,4,#^, Xiaoyan Wang^2^, Xia Peng^1,4^, Yinchun Ji^1,4^, Lu Tang^2,4^, Qichang He^2^, Danqi Chen^2,4^, Ye Yang^1,4^, Xuan Zhou^1,4^, Bing Xiong^2,4,*^, Jing Ai^1,3,4,*^

**Author affiliations:**

^1^ State Key Laboratory of Drug Research, Shanghai Institute of Materia Medica, Chinese Academy of Sciences, Shanghai 201203, China

^2^ State Key Laboratory of Chemical Biology, Shanghai Institute of Materia Medica, Chinese Academy of Sciences, Shanghai 201203, China

^3^ Shandong Laboratory of Yantai Drug Discovery, Bohai Rim Advanced Research Institute for Drug Discovery, Yantai, Shandong 264117, China

^4^ University of Chinese Academy of Sciences, No.19(A) Yuquan Road, Shijingshan District, Beijing, P.R.China 100049

^#^ These authors contributed equally to this work.

* Corresponding authors

JA, E-mail: [jai@simm.ac.cn](mailto:jai@simm.ac.cn)

BX, Email: bxiong@simm.ac.cn**Table S1** Inhibition activities of compound **2-13** on MK2.

| **No.** | **A** | **R^1^** | **R^2^** | **IC_50_ (nM)*^a^*** |
| --- | --- | --- | --- | --- |
| **2** | A1 | H | H | 728.1±98.9 |
| **3** | A2 | H | H | 30% inhibition@1 μM |
| **4** | A1 | OMe | H | 452.2±45.3 |
| **5** | A1 | H | OMe | 36.9±20.1 |
| **6** | A1 | Me | H | 89.5±15.3 |
| **7** | A1 | H | Me | 284.7±97.5 |
| **8** | A1 | CF_3_ | H | 216.9±4.8 |
| **9** | A1 | H | CF_3_ | 52.1±0.3 |
| **10** | A1 | OCF_3_ | H | 212.0±8.3 |
| **11** | A1 | H | OCF_3_ | 2.3±0.8 |
| **12** | A1 | NMe_2_ | H | 121.5±26.1 |
| **13** | A1 | H | NMe_2_ | 112.7±3.5 |
| **PF-3544022** |  |  |  | 12.3±1.3 |
| **CC-99677** |  |  |  | 143.5±0.7 |

^a^Values are the mean ± SD or inhibitory rate of two independent tests

| **Table S2** Kinase profiling of compound **11** against other 380 human protein kinases | | | | | | | |
| --- | --- | --- | --- | --- | --- | --- | --- |
| Concentration (nM)    Kinases | Inhibition rate (%) | | | | | | |
|  | 1 | 10 | | 100 | | 1000 | |
| p38-alpha/SAPK2a(h) |  | | 0 | | -3 | | -5 |
| MAPKAP-K3(h) | -10 | | 1 | | 12 | |  |
| MAPKAP-K5/PRAK(h) | -21 | | 6 | | 29 | |  |
| p70S6K(h) |  | | -9 | | -16 | | -6 |
| FGFR4(h) |  | | 0 | | -5 | | -1 |
| TTK(h) |  | | -1 | | 7 | | 43 |
| AAK1(h) |  | | -1 | | 11 | | 39 |
| Abl(h) |  | | -9 | | -5 | | 9 |
| Abl (H396P) (h) |  | | 6 | | 3 | | 1 |
| Abl (M351T)(h) |  | | 9 | | 2 | | 22 |
| Abl (Q252H) (h) |  | | -6 | | 5 | | 27 |
| Abl(T315I)(h) |  | | 6 | | 11 | | 31 |
| Abl(Y253F)(h) |  | | 0 | | 2 | | 21 |
| ACK1(h) |  | | 1 | | 2 | | 3 |
| ACTR2(h) |  | | -9 | | -11 | | -8 |
| ALK(h) |  | | 6 | | 6 | | 16 |
| ALK1(h) |  | | -16 | | -12 | | -9 |
| ALK2(h) |  | | -4 | | -6 | | 0 |
| ALK4(h) |  | | 26 | | 16 | | 6 |
| ALK6(h) |  | | -13 | | 4 | | 6 |
| Arg(h) |  | | 1 | | 2 | | 6 |
| AMPKα1(h) |  | | 6 | | 20 | | 65 |
| AMPKα2(h) |  | | 3 | | 11 | | 43 |
| A-Raf(h) |  | | 1 | | 15 | | 18 |
| ARK5(h) |  | | -2 | | 14 | | 28 |
| ASK1(h) |  | | 14 | | 33 | | 83 |
| Aurora-A(h) |  | | -1 | | 1 | | 7 |
| Aurora-B(h) |  | | 4 | | 19 | | 27 |
| Aurora-C(h) |  | | 2 | | -5 | | 6 |
| Axl(h) |  | | -21 | | -6 | | 26 |
| BIKe(h) |  | | 0 | | 18 | | 48 |
| Blk(h) |  | | 13 | | 4 | | 12 |
| BMPR2(h) |  | | 8 | | 13 | | 28 |
| Bmx(h) |  | | 1 | | -4 | | -5 |
| BRK(h) |  | | 0 | | -5 | | -7 |
| BrSK1(h) |  | | -7 | | -3 | | 17 |
| BrSK2(h) |  | | -2 | | 6 | | 26 |
| BTK(h) |  | | -4 | | 10 | | 17 |
| BTK(R28H)(h) |  | | -27 | | 6 | | 1 |
| B-Raf(h) |  | | -7 | | -7 | | 4 |
| B-Raf(V599E)(h) |  | | -6 | | -14 | | -2 |
| CaMKI(h) |  | | -3 | | -3 | | -6 |
| CaMKIß(h) |  | | -6 | | -2 | | -2 |
| CaMKIγ(h) |  | | -9 | | -14 | | -15 |
| CaMKIIα(h) |  | | -3 | | -4 | | -2 |
| CaMKIIβ(h) |  | | -1 | | 9 | | 14 |
| CaMKIIγ(h) |  | | -5 | | 6 | | 18 |
| CaMKIδ(h) |  | | -3 | | -6 | | 1 |
| CaMKIIδ(h) |  | | -7 | | 11 | | 20 |
| CaMKIV(h) |  | | 9 | | 8 | | 6 |
| CaMKK1(h) |  | | -7 | | 4 | | 11 |
| CaMKK2(h) |  | | -9 | | -1 | | 38 |
| Cdc7/cyclinB1(h) |  | | 8 | | 16 | | 22 |
| CDK1/cyclinB(h) |  | | 0 | | 10 | | 43 |
| CDK2/cyclinA(h) |  | | -5 | | 0 | | 7 |
| CDK2/cyclinE(h) |  | | -3 | | 1 | | -1 |
| CDK3/cyclinE(h) |  | | -5 | | -1 | | 0 |
| CDK4/cyclinD3(h) |  | | 3 | | 0 | | 2 |
| CDK5/p25(h) |  | | -6 | | 2 | | -2 |
| CDK5/p35(h) |  | | -7 | | -6 | | 5 |
| CDK6/cyclinD3(h) |  | | 2 | | 6 | | -3 |
| CDK7/cyclinH/MAT1(h) |  | | 5 | | -1 | | -2 |
| CDK9/cyclin T1(h) |  | | 8 | | 2 | | 6 |
| CDK12/cyclinK(h) |  | | -1 | | 1 | | 5 |
| CDK13/cyclinK(h) |  | | 1 | | 7 | | 9 |
| CDK14/cyclinY(h) |  | | -2 | | 7 | | 4 |
| CDK16/cyclinY(h) |  | | -1 | | 7 | | -13 |
| CDK17/cyclinY(h) |  | | 13 | | 19 | | 25 |
| CDK18/cyclinY(h) |  | | -5 | | 2 | | -3 |
| CDKL1(h) |  | | 26 | | 47 | | 49 |
| CDKL2(h) |  | | 31 | | 50 | | 54 |
| CDKL3(h) |  | | 52 | | 88 | | 99 |
| CDKL4(h) |  | | 50 | | 83 | | 93 |
| ChaK1(h) |  | | -7 | | 1 | | -5 |
| CHK1(h) |  | | 10 | | 6 | | 6 |
| CHK2(h) |  | | 3 | | 16 | | 55 |
| CK1α(h) |  | | 15 | | 30 | | 70 |
| CK1ε(h) |  | | -5 | | 4 | | 34 |
| CK1γ1(h) |  | | 0 | | 14 | | 56 |
| CK1γ2(h) |  | | -7 | | 29 | | 74 |
| CK1γ3(h) |  | | -13 | | 15 | | 67 |
| CK1δ(h) |  | | 3 | | 26 | | 74 |
| CK2(h) |  | | -3 | | -1 | | 0 |
| CK2α1(h) |  | | -9 | | -2 | | 9 |
| CK2α2(h) |  | | 10 | | 14 | | 26 |
| CLIK1(h) |  | | 5 | | 33 | | 65 |
| CLK1(h) |  | | -7 | | 12 | | 70 |
| CLK2(h) |  | | 1 | | 10 | | 42 |
| CLK3(h) |  | | -3 | | 3 | | 10 |
| CLK4(h) |  | | -6 | | 25 | | 76 |
| cKit(h) |  | | 1 | | -14 | | 9 |
| CRIK(h) |  | | -8 | | 6 | | 29 |
| CSK(h) |  | | -2 | | -1 | | -13 |
| c-RAF(h) |  | | -1 | | 2 | | 1 |
| cSRC(h) |  | | 1 | | 8 | | 10 |
| DAPK1(h) |  | | 2 | | 5 | | 12 |
| DAPK2(h) |  | | 8 | | 3 | | 13 |
| DCAMKL1(h) |  | | 3 | | -1 | | -1 |
| DCAMKL2(h) |  | | 4 | | 4 | | 4 |
| DCAMKL3(h) |  | | -1 | | 6 | | 26 |
| DDR1(h) |  | | 0 | | -1 | | -7 |
| DDR2(h) |  | | -7 | | -10 | | -10 |
| DMPK(h) |  | | -5 | | 1 | | 7 |
| DRAK1(h) |  | | 13 | | 41 | | 82 |
| DRAK2(h) |  | | 28 | | 80 | | 93 |
| DYRK1A(h) |  | | 20 | | 76 | | 96 |
| DYRK1B(h) |  | | 21 | | 77 | | 97 |
| DYRK2(h) |  | | 16 | | 67 | | 98 |
| DYRK3(h) |  | | 34 | | 83 | | 95 |
| eEF-2K(h) |  | | -4 | | -10 | | -27 |
| EGFR(h) |  | | 3 | | -20 | | -6 |
| EphA1(h) |  | | -2 | | -5 | | -2 |
| EphA2(h) |  | | 0 | | 3 | | -2 |
| EphA3(h) |  | | 15 | | -1 | | -10 |
| EphA4(h) |  | | -1 | | 3 | | -7 |
| EphA5(h) |  | | -1 | | 4 | | 1 |
| EphA7(h) |  | | 3 | | -3 | | -11 |
| EphA8(h) |  | | 1 | | 2 | | 1 |
| EphB2(h) |  | | 5 | | -2 | | -14 |
| EphB1(h) |  | | -17 | | -12 | | 2 |
| EphB3(h) |  | | 3 | | -12 | | -1 |
| EphB4(h) |  | | -4 | | -15 | | -4 |
| ErbB2(h) |  | | 10 | | 5 | | 1 |
| ErbB4(h) |  | | -15 | | -3 | | 6 |
| FAK(h) |  | | -10 | | -1 | | 1 |
| Fer(h) |  | | -11 | | -18 | | -11 |
| Fes(h) |  | | -7 | | -5 | | 0 |
| FGFR1(h) |  | | -2 | | 6 | | 7 |
| FGFR2(h) |  | | -22 | | -12 | | -2 |
| FGFR3(h) |  | | 2 | | 9 | | 0 |
| Fgr(h) |  | | 1 | | 0 | | 0 |
| Flt1(h) |  | | -24 | | -17 | | -11 |
| Flt3(h) |  | | -12 | | -5 | | 24 |
| Flt4(h) |  | | -8 | | 5 | | 43 |
| Fms(h) |  | | -8 | | -9 | | -6 |
| Fyn(h) |  | | 0 | | 0 | | 3 |
| GCK(h) |  | | -2 | | -9 | | 17 |
| GCN2(h) |  | | 7 | | 9 | | 5 |
| GRK1(h) |  | | -6 | | -4 | | -1 |
| GRK2(h) |  | | -6 | | -9 | | -2 |
| GRK3(h) |  | | -3 | | -8 | | -2 |
| GRK5(h) |  | | -6 | | -2 | | 0 |
| GRK6(h) |  | | -1 | | -1 | | 1 |
| GRK7(h) |  | | -4 | | -6 | | 2 |
| GSK3α(h) |  | | 15 | | -9 | | 10 |
| GSK3β(h) |  | | -10 | | -7 | | 5 |
| Haspin(h) |  | | 1 | | 11 | | 46 |
| Hck(h) |  | | -3 | | -7 | | 4 |
| HIPK1(h) |  | | -21 | | -9 | | -3 |
| HIPK2(h) |  | | -13 | | -9 | | 3 |
| HIPK3(h) |  | | -9 | | -8 | | 1 |
| HIPK4(h) |  | | -23 | | -10 | | 23 |
| HPK1(h) |  | | -8 | | -15 | | 1 |
| HRI(h) |  | | -3 | | 4 | | 7 |
| ICK(h) |  | | 3 | | 18 | | 8 |
| IGF-1R(h) |  | | -3 | | -2 | | -14 |
| IKKα(h) |  | | -15 | | -14 | | -5 |
| IKKβ(h) |  | | -15 | | -12 | | -12 |
| IKKε(h) |  | | -2 | | -1 | | 5 |
| IR(h) |  | | -9 | | -4 | | -10 |
| IRE1(h) |  | | 0 | | -2 | | 0 |
| IRR(h) |  | | 10 | | 33 | | 61 |
| IRAK1(h) |  | | -1 | | 3 | | 34 |
| IRAK4(h) |  | | -4 | | -9 | | -2 |
| Itk(h) |  | | 1 | | -2 | | 3 |
| JAK1(h) |  | | -6 | | -8 | | 1 |
| JAK2(h) |  | | -6 | | -10 | | -8 |
| JAK3(h) |  | | 8 | | 5 | | 12 |
| JNK1α1(h) |  | | 2 | | 4 | | 21 |
| JNK2α2(h) |  | | -6 | | 5 | | 23 |
| JNK3(h) |  | | 4 | | 6 | | 38 |
| KDR(h) |  | | 4 | | 5 | | 31 |
| LATS1(h) |  | | -11 | | -3 | | 1 |
| LATS2(h) |  | | 1 | | 8 | | 4 |
| Lck(h) |  | | -7 | | -5 | | -10 |
| LIMK1(h) |  | | -5 | | -2 | | -2 |
| LIMK2(h) |  | | -9 | | -8 | | -1 |
| LKB1(h) |  | | -5 | | -3 | | -6 |
| LOK(h) |  | | -2 | | 6 | | 54 |
| Lyn(h) |  | | -1 | | -11 | | -11 |
| LRRK2(h) |  | | -4 | | 17 | | 74 |
| LTK(h) |  | | -6 | | 2 | | 6 |
| MAK(h) |  | | -9 | | -1 | | -2 |
| MAPK1(h) |  | | -1 | | 0 | | 7 |
| MAPK2(h) |  | | -5 | | -7 | | 13 |
| MAP4K3(h) |  | | -8 | | -4 | | 3 |
| MAP4K4(h) |  | | -11 | | -1 | | 33 |
| MAP4K5(h) |  | | -7 | | -5 | | 12 |
| MEK1(h) |  | | -12 | | -4 | | -8 |
| MEK2(h) |  | | -4 | | 4 | | 6 |
| MARK1(h) |  | | -1 | | -12 | | -4 |
| MARK3(h) |  | | 2 | | -4 | | 5 |
| MARK4(h) |  | | -10 | | -2 | | -4 |
| MEKK2(h) |  | | -3 | | 2 | | -2 |
| MEKK3(h) |  | | -5 | | 11 | | 12 |
| MELK(h) |  | | -7 | | 5 | | 30 |
| Mer(h) |  | | -21 | | 4 | | 28 |
| Met(h) |  | | 6 | | 21 | | 62 |
| MINK(h) |  | | -10 | | 3 | | 31 |
| MKK3(h) |  | | 6 | | -2 | | -1 |
| MKK6(h) |  | | -4 | | -9 | | -15 |
| MLCK(h) |  | | -10 | | 0 | | 34 |
| MLK1(h) |  | | -3 | | 0 | | 10 |
| MLK2(h) |  | | -5 | | 2 | | 10 |
| MLK3(h) |  | | -2 | | 8 | | 43 |
| MLK4(h) |  | | -2 | | -2 | | -3 |
| Mnk2(h) |  | | 4 | | 13 | | 61 |
| MOK(h) |  | | 11 | | 7 | | 2 |
| MRCKα(h) |  | | -5 | | -3 | | -3 |
| MRCKβ(h) |  | | 8 | | 0 | | 10 |
| MRCKγ(h) |  | | -13 | | 2 | | 12 |
| MSK1(h) |  | | -11 | | -12 | | -11 |
| MSK2(h) |  | | -8 | | -5 | | -13 |
| MSSK1(h) |  | | -7 | | -4 | | 0 |
| MST1(h) |  | | -1 | | -9 | | 0 |
| MST2(h) |  | | -7 | | 0 | | 6 |
| MST3(h) |  | | -6 | | 2 | | 2 |
| MST4(h) |  | | -5 | | -8 | | 2 |
| mTOR(h) |  | | -1 | | 2 | | 7 |
| mTOR/FKBP12(h) |  | | 8 | | 12 | | 6 |
| MuSK(h) |  | | 7 | | 7 | | 7 |
| MYLK2(h) |  | | -2 | | 6 | | 29 |
| MYO3B(h) |  | | 3 | | 4 | | 16 |
| NDR1(h) |  | | -17 | | -14 | | -12 |
| NDR2(h) |  | | -5 | | -10 | | -2 |
| NEK1(h) |  | | -3 | | -1 | | 11 |
| NEK2(h) |  | | -3 | | 0 | | 11 |
| NEK3(h) |  | | -6 | | 0 | | -1 |
| NEK4(h) |  | | -2 | | 5 | | 34 |
| NEK6(h) |  | | -4 | | -5 | | -3 |
| NEK7(h) |  | | -5 | | -2 | | 3 |
| NEK9(h) |  | | -2 | | -6 | | 1 |
| NIM1(h) |  | | -4 | | -7 | | -3 |
| NEK11(h) |  | | -1 | | 0 | | -4 |
| NLK(h) |  | | 6 | | 16 | | 42 |
| NUAK2(h) |  | | -16 | | -8 | | 8 |
| OSR1(h) |  | | -1 | | 5 | | 11 |
| PAK1(h) |  | | -10 | | -7 | | -5 |
| PAK2(h) |  | | -3 | | -5 | | -3 |
| PAK4(h) |  | | 2 | | -2 | | 2 |
| PAK3(h) |  | | -4 | | 0 | | -3 |
| PAK5(h) |  | | -2 | | -2 | | -2 |
| PAK6(h) |  | | -2 | | -5 | | -2 |
| PAR-1Bα(h) |  | | -6 | | -2 | | -3 |
| PASK(h) |  | | 13 | | 4 | | 12 |
| PEK(h) |  | | -1 | | -9 | | 2 |
| PDGFRα(h) |  | | -5 | | -3 | | -2 |
| PDGFRβ(h) |  | | 0 | | -16 | | -11 |
| PDHK2(h) |  | | -8 | | 6 | | 0 |
| PDHK4(h) |  | | -5 | | -7 | | -19 |
| PDK1(h) |  | | -9 | | -2 | | 3 |
| PhKγ1(h) |  | | -3 | | -3 | | 2 |
| PhKγ2(h) |  | | 8 | | 1 | | 2 |
| Pim-1(h) |  | | -12 | | 12 | | 41 |
| Pim-2(h) |  | | -3 | | 5 | | 6 |
| Pim-3(h) |  | | -1 | | 16 | | 59 |
| PKA(h) |  | | -7 | | -7 | | -6 |
| PKAcβ(h) |  | | -11 | | -4 | | 5 |
| PKBα(h) |  | | 2 | | 4 | | 14 |
| PKBβ(h) |  | | 1 | | 3 | | 0 |
| PKBγ(h) |  | | 0 | | 3 | | 9 |
| PKCα(h) |  | | 5 | | 14 | | 6 |
| PKCβI(h) |  | | 3 | | 1 | | -13 |
| PKCβII(h) |  | | -1 | | 4 | | 6 |
| PKCγ(h) |  | | 7 | | 5 | | 14 |
| PKCδ(h) |  | | 1 | | 14 | | 9 |
| PKCε(h) |  | | 1 | | 12 | | 13 |
| PKCη(h) |  | | 1 | | 10 | | 9 |
| PKCι(h) |  | | 0 | | 5 | | 8 |
| PKCμ(h) |  | | -6 | | 13 | | 45 |
| PKCθ(h) |  | | 6 | | 0 | | 4 |
| PKCζ(h) |  | | -5 | | -4 | | -8 |
| PKD2(h) |  | | 0 | | 8 | | 47 |
| PKD3(h) |  | | 4 | | 15 | | 55 |
| PKG1α(h) |  | | 1 | | -4 | | 8 |
| PKG1β(h) |  | | 7 | | 10 | | 5 |
| PKR(h) |  | | 3 | | 3 | | 9 |
| Plk1(h) |  | | 13 | | 15 | | 13 |
| Plk3(h) |  | | 10 | | 6 | | 15 |
| Plk4(h) |  | | 9 | | 4 | | 33 |
| PRKG2(h) |  | | 7 | | 19 | | 35 |
| PRK1(h) |  | | -3 | | 6 | | 4 |
| PRK2(h) |  | | -2 | | 3 | | 5 |
| PrKX(h) |  | | 3 | | 12 | | 11 |
| PRP4(h) |  | | -1 | | -3 | | 12 |
| PTK5(h) |  | | -7 | | 6 | | 5 |
| Pyk2(h) |  | | -11 | | -5 | | 6 |
| Ret(h) |  | | 12 | | 2 | | 1 |
| RIPK1(h) |  | | -7 | | -3 | | 3 |
| RIPK2(h) |  | | 11 | | 46 | | 88 |
| ROCK-I(h) |  | | 2 | | -1 | | -7 |
| ROCK-II(h) |  | | -3 | | 0 | | -6 |
| Ron(h) |  | | -4 | | 26 | | 77 |
| Ros(h) |  | | 1 | | 1 | | 6 |
| Rse(h) |  | | 7 | | -3 | | -5 |
| Rsk1(h) |  | | -2 | | -3 | | -7 |
| Rsk2(h) |  | | -6 | | -11 | | -3 |
| Rsk3(h) |  | | -9 | | 14 | | 16 |
| Rsk4(h) |  | | -9 | | -4 | | -13 |
| p38-beta/SAPK2b(h) |  | | -7 | | -11 | | -4 |
| p38-gamma/SAPK3(h) |  | | 5 | | 2 | | 20 |
| p38-delta/SAPK4(h) |  | | -3 | | -6 | | 8 |
| SBK1(h) |  | | -1 | | -8 | | 3 |
| SGK(h) |  | | 0 | | 7 | | 1 |
| SGK2(h) |  | | -10 | | -7 | | 0 |
| SGK3(h) |  | | -8 | | 2 | | -4 |
| SIK(h) |  | | 2 | | 4 | | -3 |
| SIK2(h) |  | | 2 | | 4 | | 8 |
| SIK3(h) |  | | 6 | | 1 | | 12 |
| SLK(h) |  | | 1 | | 4 | | 4 |
| Snk(h) |  | | 0 | | 10 | | 15 |
| SNRK(h) |  | | 4 | | 4 | | 9 |
| Src(1-530)(h) |  | | 6 | | 5 | | -1 |
| SRMS(h) |  | | 1 | | 10 | | 7 |
| SRPK1(h) |  | | 6 | | 13 | | 22 |
| SRPK2(h) |  | | 7 | | -1 | | 6 |
| STK16(h) |  | | 0 | | -3 | | 11 |
| STK25(h) |  | | -4 | | 1 | | 0 |
| STK32A(h) |  | | 3 | | 10 | | 7 |
| STK32B(h) |  | | -9 | | -5 | | -3 |
| STK32C(h) |  | | 0 | | 4 | | 1 |
| STK33(h) |  | | 8 | | 18 | | 64 |
| STK39(h) |  | | -10 | | -4 | | 5 |
| Syk(h) |  | | 5 | | -15 | | -9 |
| TAF1L(h) |  | | 51 | | 74 | | 80 |
| TAK1(h) |  | | 9 | | 2 | | 47 |
| TAO1(h) |  | | 3 | | 6 | | 11 |
| TAO2(h) |  | | -7 | | 1 | | 0 |
| TAO3(h) |  | | -4 | | 0 | | 7 |
| TBK1(h) |  | | 1 | | -13 | | -7 |
| Tec(h) activated |  | | 8 | | 11 | | 17 |
| TGFBR1(h) |  | | -7 | | -2 | | -4 |
| TGFBR2(h) |  | | -11 | | 2 | | 6 |
| Tie2 (h) |  | | 0 | | 10 | | 24 |
| TLK1(h) |  | | 13 | | 0 | | -5 |
| TLK2(h) |  | | 13 | | 1 | | -2 |
| TNIK(h) |  | | -12 | | 9 | | 42 |
| TRB2(h) |  | | 29 | | 58 | | 85 |
| TrkA(h) |  | | -6 | | -1 | | 15 |
| TrkB(h) |  | | -6 | | -9 | | -20 |
| TrkC(h) |  | | -3 | | 4 | | -3 |
| TSSK1(h) |  | | 1 | | 2 | | 7 |
| TSSK2(h) |  | | -7 | | -5 | | -5 |
| TSSK3(h) |  | | 0 | | 5 | | -2 |
| TSSK4(h) |  | | 3 | | 5 | | 2 |
| TTBK1(h) |  | | -2 | | -5 | | 3 |
| TTBK2(h) |  | | -1 | | -3 | | 3 |
| Txk(h) |  | | -3 | | 1 | | -1 |
| TYK2(h) |  | | 1 | | 10 | | 14 |
| ULK1(h) |  | | 6 | | -8 | | -3 |
| ULK2(h) |  | | 6 | | 6 | | 5 |
| ULK3(h) |  | | 8 | | 3 | | -4 |
| VRK1(h) |  | | 1 | | 1 | | 5 |
| VRK2(h) |  | | 7 | | 8 | | 7 |
| Wee1(h) |  | | -11 | | -11 | | -4 |
| Wee1B(h) |  | | -2 | | -4 | | 3 |
| WNK1(h) |  | | 6 | | 2 | | 4 |
| WNK2(h) |  | | 11 | | 19 | | 17 |
| WNK3(h) |  | | 0 | | 7 | | 9 |
| WNK4(h) |  | | 6 | | 6 | | 3 |
| Yes(h) |  | | -2 | | 0 | | -3 |
| ZAK(h) |  | | -3 | | -1 | | 1 |
| ZAP-70(h) |  | | 8 | | 0 | | -13 |
| ZIPK(h) |  | | 4 | | 8 | | 15 |
| ATM(h) |  | | -3 | | -6 | | -9 |
| ATR/ATRIP(h) |  | | -15 | | -17 | | -20 |
| DNA-PK(h) |  | | -1 | | 2 | | 3 |
| PI3 Kinase (p110β/p85α)(h) |  | | -1 | | -1 | | -1 |
| PI3 Kinase (p120γ)(h) |  | | 1 | | -1 | | 3 |
| PI3 Kinase (p110δ/p85α)(h) |  | | -3 | | -1 | | 2 |
| PI3 Kinase (p110α/p85α)(h) |  | | -1 | | 0 | | -1 |
| PI3 Kinase (p110α(E542K)/p85α)(h) |  | | -3 | | -2 | | -2 |
| PI3 Kinase (p110α(H1047R)/p85α)(h) |  | | 0 | | 0 | | 2 |
| PI3 Kinase (p110α(E545K)/p85α)(h) |  | | 2 | | 2 | | 4 |
| PI3 Kinase (p110α/p65α)(h) |  | | 3 | | 0 | | 1 |
| PI3KC2α(h) |  | | -4 | | -2 | | -5 |
| PI3KC2γ(h) |  | | 6 | | 13 | | 12 |
| PIP4K2α(h) |  | | -1 | | -3 | | -1 |
| PIP5K1α(h) |  | | -4 | | -3 | | -2 |
| PIP5K1γ(h) |  | | 0 | | -1 | | 3 |

**Table S3** Pharmacokinetic parameters of compound **11**.

| **ip. (10 mg/kg)** | **Mean** ± SD |
| --- | --- |
| **T_1/2_ (h)** | 0.38±0.034 |
| **T_max_ (h)** | 0.05±0.00 |
| **C_max_ (ng/mL)** | 125.87±46.0 |
| **AUC_last_ (h*ng/mL)** | 44.49±21.4 |
| **AUC_inf_obc_ (h*ng/mL)** | 45.19±21.3 |
| **MRT_inf_obs_ (h)** | 1.64±2.08 |

**Table S4** The cell cytotoxicity of compound **11.**

| Cell type | | Cell line | IC_50_ (μM)  mean ± SD |
| --- | --- | --- | --- |
| normal cell line | mouse mononuclear macrophage | Raw264.7 | 31.7 ± 2.9 |
|  | human monocyte | THP-1 | 34.7 ± 0.4 |
|  | human embryo kidney | HEK293 | 29.9 ± 7.2 |
| tumor cell line | murine colorectal carcinoma | MC38 | 48.3 ± 10.5 |
|  |  | CT26 | 47.2 ± 2.4 |
|  | human non-small cell lung carcinoma | HCC44 | 32.3 ± 3.3 |
|  |  | H1437 | 33.4 ± 0.6 |
|  | human pancreatic carcinoma | MIA Paca-2 | 47.8 ± 2.8 |
|  |  | PANC-1 | 35.3 ± 1.4 |

**Table S5** List of primers for qPCR

| Primers | Sequence (5′–3′) |
| --- | --- |
| Mouse-β-actin forward | ATCACTATTGGCAACGAGCGGTTC |
| Mouse-β-actin reverse | CAGCACTGTGTTGGCATAGAGGTC |
| Human-β-actin forward | TTGCTGATCCACATCTGCT |
| Human-β-actin reverse | GACAGGATGCAGAAGGAGAT |
| Mouse-TNF-α forward | TTCTGTCTACTGAACTTCGGGGTGATCGGTCC |
| Mouse-TNF-α reverse | GTATGAGATAGCAAATCGGCTGACGGTGTGGG |
| Human-TNF-α forward | GAGGCCAAGCCCTGGTATG |
| Human-TNF-α reverse | CGGGCCGATTGATCTCAGC |
| Mouse-IL-1β forward | CAACCAACAAGTGATATTCTCCATG |
| Mouse-IL-1β reverse | GATCCACACTCTCCAGCTGCA |
| Human-IL-1β forward | GCCAGTGAAATGATGGCTTATT |
| Human-IL-1β reverse | AGGAGCACTTCATCTGTTTAGG |
| Mouse-IL-6 forward | CTCCCAACAGACCTGTCTATAC |
| Mouse-IL-6 reverse | CCATTGCACAACTCTTTTCTCA |
| Human-IL-6 forward | CACTGGTCTTTTGGAGTTTGAG |
| Human-IL-6 reverse | GGACTTTTGTACTCATCTGCAC |
| Mouse-ARG-1 forward | CATATCTGCCAAAGACATCGTG |
| Mouse-ARG-1 reverse | GACATCAAAGCTCAGGTGAATC |
| Human-ARG-1 forward | TGGACAGACTAGGAATTGGCA |
| Human-ARG-1 reverse | CCAGTCCGTCAACATCAAAACT |
| Mouse-MRC1 forward | CCTATGAAAATTGGGCTTACGG |
| Mouse-MRC1 reverse | CTGACAAATCCAGTTGTTGAGG |
| Human-MRC1 forward | TCCGACCCTTCCTTGACTAATCCTC |
| Human-MRC1 reverse | AGTATGTCTCCGCTTCATGCCATTG |
| Mouse-VEGF forward | GCACATAGAGAGAATGAGCTTCC |
| Mouse-VEGF reverse | CTCCGCTCTGAACAAGGCT |
| Human-VEGF forward | GGGCTGCTGCAATGACGA |
| Human-VEGF reverse | CCTATGTGCTGGCCTTGGT |

**Table S6** List of sequence of siRNA

| Name | Sequence (5′–3′) |
| --- | --- |
| Human-MK2 siRNA S1 sense | GGAUCAUGCAAUCAACAAATT |
| Human-MK2 siRNA S1 antisense | UUUGUUGAUUGCAUGAUCCAA |
| Human-MK2 siRNA S2 sense | UGACCAUCACCGAGUUUAUdTdT |
| Human-MK2 siRNA S2 antisense | AUAAACUCGGUGAUGGUCAdTdT |
| Human-MK2 siRNA NC | GUUGAGAGAUAUUAGAGUU |

**Figure S1** Representative MK2 inhibitors.


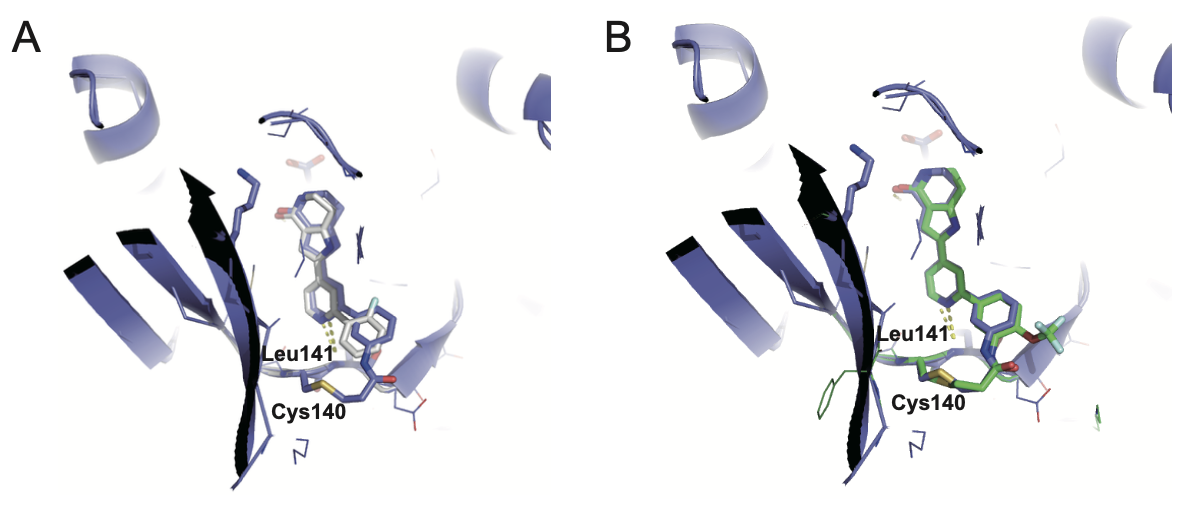


**Figure S2** Docking study on compounds **2** and **11** with the crystal structure of MK2 (PDB ID: 2P3G). (A) Proposed covalent binding mode of compound **2** (purple), and superimposition with the ligand (compound **1**, gray) of 2P3G. (B) Superimposition of compound **2** (purple) and **11** (green).


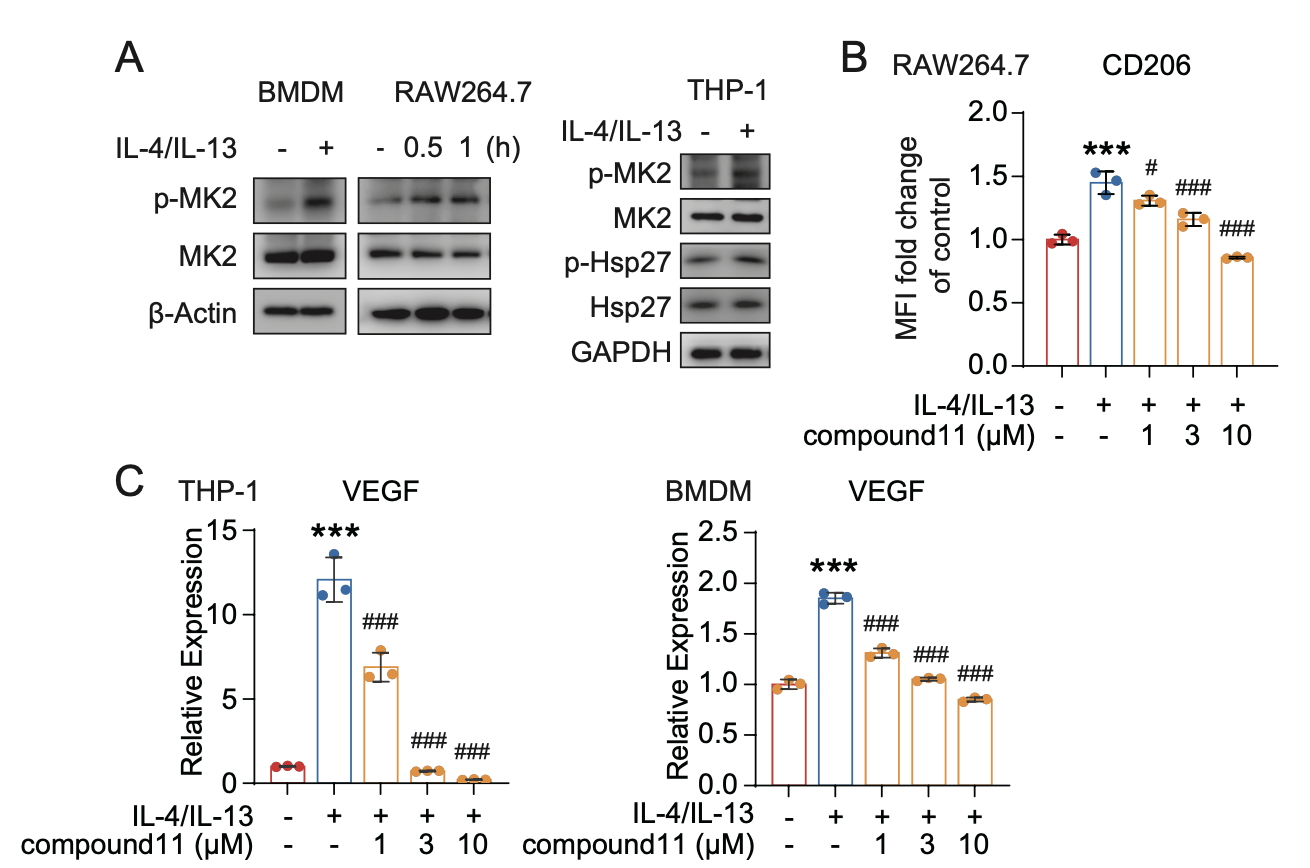


**Figure S3** Inhibition of pro-tumorigenic M2-like polarization in macrophages by compound **11**. (A) Illustrates the treatment effects on various macrophage cells using Western blot analysis post IL-4/IL-13 exposure (30 minutes or the specified time period). (B) Examines CD206 expression in RAW264.7 cells via flow cytometry post 48hours treatment of compound **11** and IL-4/IL-13. (C) RT-PCR analyses demonstrate the suppression of VEGF expression in THP-1 cells (left) and bone marrow-derived macrophages (BMDMs) (right) treated with IL-4/IL-13 alone or in combination with compound **11** for 12 hours. IL-4/IL-13 (20 ng/mL) served as the stimulation control, while untreated cells were the baseline control. The data, presented as mean ± SD from triplicates, show significant reductions in VEGF transcription and CD206 expression, with statistical analysis conducted via one-way ANOVA (*^#^P* < 0.05; *^###^P* < 0.001 vs. stimulation control; *^***^P* < 0.001 vs. baseline control).


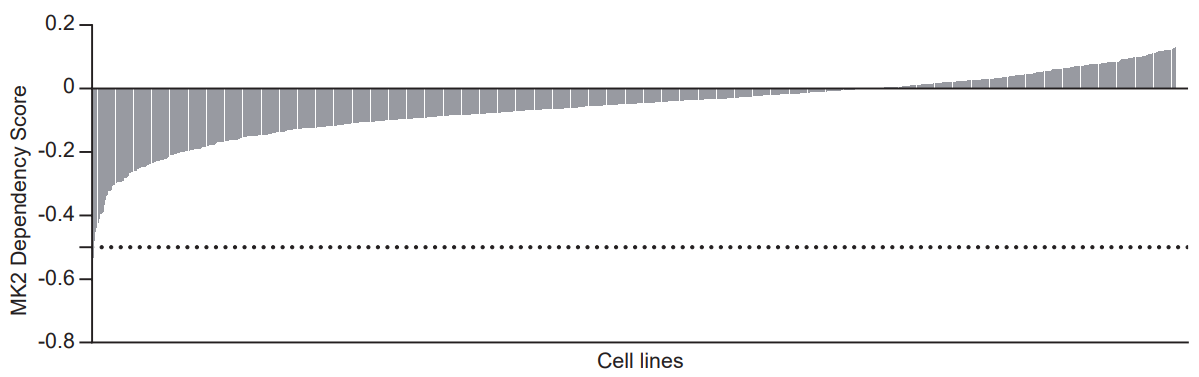


**Figure S4** MK2 Gene Dependency Across Tumor Cell Lines According to the DepMap Database. The x-axis lists different tumor cell lines, while the y-axis quantifies the MK2 dependency score. Lower scores suggest a higher necessity for the MK2 gene within each cell line, with scores below -0.5 marking the gene as essential for the cell line's survival. A score of 0 indicates non-essentiality of the gene.


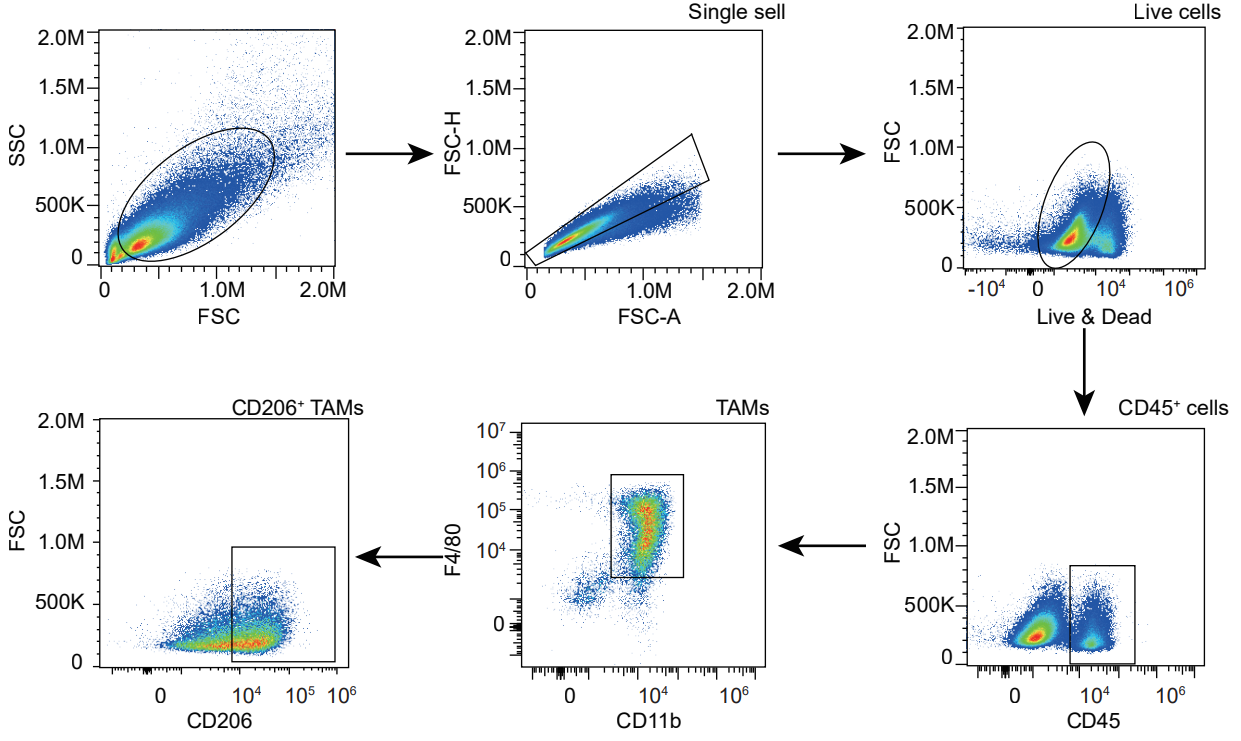


**Figure S5** The gating strategy for tumor-infiltrating immune cells.

**Supplemental methods：**

*Docking study*

Computational modeling of compounds **2** and **11** bound to MK2 was performed using the software Schrödinger. Briefly, the cocrystal structure of MK2 (PDB ID: 2P3G) was downloaded from PDB database, and Protein Preparation Wizard module was adopted to add the hydrogen atoms and refine the structure with OPLS3 force field, and saved as protein model. The ligands (**2** and **11**) were processed by LigPrep module to obtain the low energy conformations. Then, covalent molecular docking was performed by designating the residue Cys140 as the protein anchor point and Michael addition as the reaction type. After the docking, the binding conformation with the best energy was selected for visualization and interaction analysis.

*Cell inoculation number in* *cellular experiments*

In cellular experiments, cells are seeded into either 6-well or 96-well plates and incubated overnight, proceeding with treatments as described in the figure legends or methods section. The cell inoculation numbers are outlined as follows: For assays measuring cell viability, between 3,000 to 5,000 cells are seeded per well in 96-well plates. For other cellular assays, the seeding density ranges from 5×10^5^ to 1×10^6^ cells per well in 6-well plates.

*Cell viability assay*

Cells was plated into 96-well plates and incubated overnight prior to introduction of varying concentrations of compounds or control solvent. Following a 72-hour exposure period, cell viability was determined using a Cell Counting Kit-8 assay (Life-iLab, China). Dose-response curves were fitted using a four-parameter method facilitated by SoftMax Pro, which was subsequently used for the computation of IC_50_ values.

*The sample preparation and the analysis of mouse pharmacokinetics study*

10 mL of plasma was mixed with 100 mL of methanol: acetonitrile solvent (1:1, v/v) with internal standard, and the mixture was thoroughly vortexed. After centrifugation for 5 minutes, 20 mL of the supernatant was combined with 20 mL of water for analysis. Samples were analyzed by Xevo TQ-S triple quadrupole mass spectrometer. The ACQUITY UPLC BEH C^18^ (1.7 µm, 2.0mm_ 50 mm, Waters, USA) was adopted for the analysis. Gradient elution was employed using a 5 mM ammonium acetate aqueous solution containing 0.1% formic acid, along with acetonitrile containing 0.1% formic acid. Following the analysis of compound concentrations, Phoenix WinNonlin (CERTARA, USA) was utilized to calculate the value of AUC_last_, AUCINF_inf_obs_ and MRTINF_inf_obs_ from time-concentration curves in each animal. C_max_ denotes the peak plasma concentration, while T_max_ signifies the time taken to achieve the peak concentration.

*Administration of inhibitors and tumor measurement in antitumor in vivo evaluation*

The mice then received intraperitoneal injections of either the vehicle control or compound 11 (at dosages of 25 or 50 mg/kg) twice daily for 10 days. Tumor size was gauged every three days with a microcaliper, and the tumor volume (TV) was estimated using the formula: TV = (length × width^2^)/2. Additionally, the individual Tumor Growth Inhibition value (TGI) was assessed on the last day of the experiment for mice treated with the drug in comparison to those treated with the vehicle. The formula used was 100 × {1 - [(V_Treated Final day_ -V_Treated Day 0_)/(V_Control Final day_ -V_Control Day 0_)].

*Synthesis*

**General information**

^1^H NMR (400MHz) spectra were recorded with a Varian Mercury-400 High Performance Digital FT-NMR spectrometer with tetramethylsilane (TMS) as an internal standard. ^13^C NMR (100 MHz or 125 MHz) spectra were recorded by using a Varian Mercury-400 High Performance Digital FT-NMR spectrometer or Varian Mercury-500 High Performance Digital FT-NMR spectrometer. Abbreviations for peak patterns in NMR spectra: s =singlet, d =doublet, and m=multiplet. Low-resolution mass spectra were obtained with a Finnigan LCQ Deca XP mass spectrometer using a CAPCELL PAK C18 (50mm×2.0mm, 5 µM) or an Agilent ZORBAX Eclipse XDB C18 (50mm×2.1mm,5 µM) in positive or negative elec-trospray mode. High-resolution mass spectra were recorded by using a Finnigan MAT-95 mass spectrometer or an Agilent technology 6224 TOF mass spectrometer.TLC analysis was carried out with glass precoated silica gel GF254 plates. TLC spots were visualized under UV light. All solvents and reagents were used directly as obtained commercially unless otherwise noted. All air and moisture sensitive reactions were carried out under an atmosphere of dry Argon with heat-dried glassware and standard syringe techniques. CC-99677 (HY-139504) and PF-3644022 (HY-107427) were purchased from MedChemExpress (Shanghai, China).

**Scheme S1** Synthesis route of compound **2-13**

**Reagents and conditions:** (a) Br_2_, HBr, AcOH, 50 ℃, overnight; (b) Piperidine-2,4-dione, AcONH_4_, dry EtOH, r.t., 5 h; (c) Corresponding substituted 3-(4,4,5,5-tetramethyl-1,3,2-dioxaborolan-2-yl) aniline, Cs_2_CO_3_, Pd (PPh_3_)_4_, DMF: H_2_O (10: 1), N_2_, 80 ℃, 18 h; (d) Acrylyl chloride or propionyl chloride, DIEA, THF, r.t., 4 h.

General procedure for compound **2-13**.

**m4**-**m14** were synthesized according to the methods described by Anderson *et al* (*J. Med. Chem.* 2007, 50, 2647–2654).

1-(2-Chloropyridin-4-yl)ethan-1-one (3.00 g, 19.28 mmol) was dissolved in 15 mL AcOH, followed by the addition of HBr 7.5 mL. Br_2_ (3.08 g, 19.28 mmol) in 3 mL AcOH was added dropwise to the mixture at 0 ℃. The mixture was stirred at 50 ℃ for 5 h. After the reaction was complete, 15 mL ether was added and then white precipitate was formed. The solid was filtered, washed with ether for three times and then dried at 40 ℃ to get the intermediate **m2** (4.23 g, yield 93.6%). **m2** (3.00 g, 12.79 mmol) was dissolved in 30 mL EtOH, then piperidine-2,4-dione (1.59g, 14.07mmol) and AcONH_4_ (3.94 g, 51.18 mmol) were added. The mixture was stirred at room temperature for 14 h. After completion, the mixture was added 15 mL H_2_O and stirred for 20 min. The resulting precipitate was filtered, washed with H_2_O and dried at 50 ℃ to obtain the white solid intermediate **m3** (1.48 g, yield 46.7%). To a resealable vial was added intermediate **m3** (1 eq.), corresponding substituted 3-(4,4,5,5-tetramethyl-1,3,2-dioxaborolan-2-yl)aniline (1.1 eq.), Cs_2_CO_3_ (2 eq.) and the solution (N,N-dimethylformamide : H_2_O=10:1). The mixture was degassed with N_2_ for 5 min and then added Pd(PPh_3_)_4_ (0.05 eq.). The vial was sealed and heated to 80 ℃ for 18 h. After completion, the mixture was diluted with H_2_O and extracted with ethyl acetate for three times. The organic layer was combined, washed with brine, dried with Na_2_SO_4_ and concentrated. The crude product was purified by silica gel column chromatography to afford **m4**-**m14** (yield 34.8%-63.3%). The intermediate **m4**-**m14** (1 eq.) was dissolved in dry tetrahydrofuran and added N,N-diisopropylethylamine (4 eq.). Acrylyl chloride or propionyl chloride (1 eq.) was added dropwise at 0 ℃. The mixture was stirred at room temperature for 2 h. After completion, the reaction was quenched with methanol and concentrated. The crude product was purified by silica gel column chromatography to get the corresponding final product **2-13** (yield 17.8%-33.5%).

*N*-(3-(4-(4-oxo-4,5,6,7-tetrahydro-1H-pyrrolo[3,2-c]pyridin-2-yl)pyridin-2-yl)phenyl)acrylamide (**2**). Yellow solid. MS (ESI) m/z: 359.44 [M+H]^+^. ^1^H NMR (400 MHz, DMSO-*d*_6_) *δ* 12.05 (s, 1H), 10.32 (s, 1H), 8.57 (d, *J* = 5.3 Hz, 1H), 8.43 (s, 1H), 8.18 (s, 1H), 7.85 (d, *J* = 7.9 Hz, 2H), 7.60 (d, *J* = 5.3 Hz, 1H), 7.48 (t, *J* = 7.9 Hz, 1H), 7.17 – 7.12 (m, 1H), 7.10 (s, 1H), 6.54 – 6.43 (m, 1H), 6.30 (d, *J* = 17.8 Hz, 1H), 5.79 (d, *J* = 10.1 Hz, 1H), 3.47 – 3.39 (m, 2H), 2.88 (t, *J* = 7.0 Hz, 2H).

*N*-(3-(4-(4-oxo-4,5,6,7-tetrahydro-1H-pyrrolo[3,2-c]pyridin-2-yl)pyridin-2-yl)phenyl)propionamide (**3**). Yellow solid. MS (ESI) m/z: 361.37 [M+H]^+^. HRMS m/z (ESI) found 361.1657 (M+H)^+^, C_21_H_21_N_4_O_2_^+^ calcd for 361.1659.^1^H NMR (400 MHz, DMSO-*d*_6_) *δ* 12.01 (s, 1H), 10.00 (s, 1H), 8.74 – 8.46 (m, 1H), 8.34 (s, 1H), 8.15 (s, 1H), 7.77 (d, *J* = 7.2 Hz, 1H), 7.59 (s, 1H), 7.42 (s, 1H), 7.21 – 7.03 (m, 2H), 3.58 – 3.39 (m, 2H), 2.96 – 2.78 (m, 2H), 2.40 – 2.27 (m, 2H), 1.17 – 1.01 (m, 3H).

*N*-(3-methoxy-5-(4-(4-oxo-4,5,6,7-tetrahydro-1*H*-pyrrolo[3,2-*c*]pyridin-2-yl)pyridin-2-yl)phenyl)acrylamide (**4**). Yellow solid. MS (ESI) m/z: 389.44 [M+H]^+^. HRMS m/z (ESI) found 389.1603 (M+H)^+^, C_22_H_21_N_4_O_3_^+^ calcd for 389.1608.  ^1^H-NMR (400 MHz, Methanol-*d*_4_) *δ* 8.30 – 8.23 (m, 1H), 8.16 (d, *J* = 7.4 Hz, 1H), 7.75 (dt, *J* = 6.8, 2.6 Hz, 1H), 7.58 (dt, *J* = 5.2, 3.4 Hz, 2H), 7.36 (d, *J* = 2.8 Hz, 1H), 7.20 (dd, *J* = 7.4, 2.8 Hz, 1H), 6.52 (dd, *J* = 17.0, 9.7 Hz, 1H), 6.44 (dd, *J* = 17.0, 2.2 Hz, 1H), 5.85 (dd, *J* = 9.7, 2.2 Hz, 1H), 4.31 – 4.25 (m, 2H), 3.55 (ddd, *J* = 14.1, 10.9, 3.2 Hz, 2H), 3.44 – 3.40 (m, 2H), 2.27 – 2.23 (m, 2H), 1.76 – 1.70 (m, 2H), 1.65 – 1.54 (m, 2H).

*N*-(2-methoxy-5-(4-(4-oxo-4,5,6,7-tetra hydro-1*H*-pyrrolo[3,2-*c*]pyridin-2-yl)pyridin-2-yl)phenyl)acrylamide (**5**). Yellow solid. MS (ESI) m/z: 389.14 [M+H]^+^. HRMS m/z (ESI) found 389.1608 (M+H)^+^, C_22_H_21_N_4_O_3_^+^ calcd for 389.1608.  ^1^H-NMR (400 MHz, DMSO-*d*_6_) *δ* 12.59 (s, 1H), 9.65 (s, 1H), 8.73 (s, 1H), 8.56 (d, *J* = 5.9 Hz, 1H), 8.34 (s, 1H), 7.95 (dd, *J* = 8.7, 2.3 Hz, 1H), 7.86 (s, 1H), 7.44 (s, 1H), 7.29 (d, *J* = 8.8 Hz, 1H), 7.21 (s, 1H), 6.75 (dd, *J* = 17.0, 10.2 Hz, 1H), 6.29 (d, *J* = 17.0 Hz, 1H), 5.78 (d, *J* = 10.3 Hz, 1H), 3.97 (s, 3H), 2.93 (t, *J* = 6.8 Hz, 2H).

*N*-(3-methyl-5-(4-(4-oxo-4,5,6,7-tetrahydro-1*H*-pyrrolo[3,2-*c*]pyridin-2-yl)pyridin-2-yl)phenyl)acrylamide (**6**). Yellow solid. MS (ESI) m/z: 373.38 [M+H]^+^. HRMS m/z (ESI) found 373.1659 (M+H)^+^, C_22_H_21_N_4_O_2_^+^ calcd for 373.1659.  ^1^H-NMR (400 MHz, DMSO-*d*_6_) *δ* 12.15 (s, 1H), 10.31 (s, 1H), 9.10 (s, 1H), 8.56 (d, *J* = 5.2 Hz, 1H), 8.24 (s, 1H), 8.17 (s, 1H), 7.70 (d, *J* = 8.1 Hz, 2H), 7.62 – 7.58 (m, 1H), 7.16 (d, *J* = 2.3 Hz, 1H), 7.10 (s, 1H), 6.50 (dd, *J* = 17.0, 10.1 Hz, 1H), 6.29 (dd, *J* = 16.8, 2.0 Hz, 1H), 5.77 (dd, *J* = 10.1, 2.1 Hz, 1H), 3.44 (s, 3H), 2.89 (t, *J* = 6.8 Hz, 2H), 2.41 (s, 3H).

*N*-(2-methyl-5-(4-(4-oxo-4,5,6,7-tetrahydro-1*H*-pyrrolo[3,2-*c*]pyridin-2-yl)pyridin-2-yl)phenyl)acrylamide (**7**). Yellow solid. MS (ESI) m/z: 373.38 [M+H]^+^. HRMS m/z (ESI) found 373.1657 (M+H)^+^, C_22_H_21_N_4_O_2_^+^ calcd for 373.1659. ^1^H-NMR (400 MHz, Methanol-*d*_4_) *δ* 8.51 (s, 1H), 8.12 (d, *J* = 15.8 Hz, 2H), 7.75 (dd, *J* = 24.6, 6.9 Hz, 2H), 7.49 (d, *J* = 7.9 Hz, 1H), 7.30 (s, 1H), 6.61 (dd, *J* = 16.8, 9.9 Hz, 1H), 6.46 (d, *J* = 17.0 Hz, 1H), 5.87 (d, *J* = 10.1 Hz, 1H), 3.62 (t, *J* = 7.0 Hz, 2H), 3.01 (t, *J* = 7.1 Hz, 2H), 2.39 (s, 3H).

*N*-(3-(4-(4-oxo-4,5,6,7-tetrahydro-1*H*-pyrrolo[3,2-*c*]pyridin-2-yl)pyridin-2-yl)-5(trifluo romethyl)phenyl)acrylamide (**8**). Yellow solid. MS (ESI) m/z: 427.38 [M+H]^+^. HRMS m/z (ESI) found 427.1363 (M+H)^+^, C_22_H_18_F_3_N_4_O_2_^+^ calcd for 427.1376. ^1^H-NMR (400 MHz, Methanol-*d*_4_) *δ* 8.56 (d, *J* = 5.3 Hz, 1H), 8.48 (s, 1H), 8.20 (s, 1H), 8.06 (d, *J* = 11.4 Hz, 2H), 7.60 (dd, *J* = 5.3, 1.7 Hz, 1H), 7.16 (s, 1H), 6.55 – 6.43 (m, 2H), 5.87 (dd, *J* = 9.0, 2.8 Hz, 1H), 3.61 (t, *J* = 7.0 Hz, 2H), 2.99 (t, *J* = 7.0 Hz, 2H).

*N*-(5-(4-(4-oxo-4,5,6,7-tetrahydro-1H-pyrrolo[3,2-c]pyridin-2-yl)pyridin-2-yl)-2-(trifluoromethyl)phenyl)acrylamide (**9**). Yellow solid. MS (ESI) m/z: 427.40 [M+H]^+^. ^1^H-NMR (400 MHz, Methanol-*d*_4_) *δ* 8.61 (d, *J* = 5.9 Hz, 1H), 8.09 (d, *J* = 8.2 Hz, 1H), 7.99 (d, *J* = 8.3 Hz, 1H), 7.86 (d, *J* = 6.3 Hz, 1H), 7.56 (d, *J* = 6.4 Hz, 1H), 7.40 (s, 1H), 7.24 (d, *J* = 6.5 Hz, 1H), 6.72 – 6.64 (m, 1H), 6.63 – 6.56 (m, 2H), 6.47 (d, *J* = 16.5 Hz, 2H), 3.25 (d, *J* = 7.4 Hz, 2H), 2.20 (d, *J* = 7.6 Hz, 2H).

*N*-(3-(4-(4-oxo-4,5,6,7-tetrahydro-1H-pyrrolo[3,2-c]pyridin-2-yl)pyridin-2-yl)-5-(trifluoromethoxy)phenyl)acrylamide (**10**). Yellow solid. MS (ESI) m/z: 443.34 [M+H]^+^. HRMS m/z (ESI) found 443.1323 (M+H)^+^, C_22_H_18_F_3_N_4_O_3_^+^ calcd for 443.1326. ^1^H NMR (400 MHz, DMSO-*d*_6_) *δ* 12.02 (s, 1H), 10.61 (s, 1H), 8.60 (d, *J* = 5.3 Hz, 1H), 8.40 (t, *J* = 1.7 Hz, 1H), 8.24 (s, 1H), 8.04 (s, 1H), 7.86 (s, 1H), 7.65 (dd, *J* = 5.3, 1.6 Hz, 1H), 7.23 (d, *J* = 2.2 Hz, 1H), 7.08 (s, 1H), 6.53 – 6.41 (m, 1H), 6.38 – 6.29 (m, 1H), 5.84 (dd, *J* = 9.9, 2.1 Hz, 1H), 3.44 (dt, *J* = 6.8, 4.0 Hz, 2H), 2.89 (t, *J* = 6.9 Hz, 2H). ^13^C NMR (151 MHz, DMSO-*d*_6_) δ 165.28, 164.12, 154.90, 150.49, 149.51, 141.63, 141.34, 140.72, 139.64, 131.91, 128.92, 128.32, 117.56, 116.65, 116.28, 114.48, 114.20, 112.20, 107.63, 40.59, 22.37.

*N*-(5-(4-(4-oxo-4,5,6,7-tetrahydro-1H-pyrrolo[3,2-c]pyridin-2-yl)pyridin-2-yl)-2-(trifluoromethoxy)phenyl)acrylamide (**11**). Yellow solid. MS (ESI) m/z: 443.40 [M+H]^+^. HRMS m/z (ESI) found 443.1326 (M+H)^+^, C_22_H_18_F_3_N_4_O_3_^+^ calcd for 443.1326. ^1^H-NMR (400 MHz, Methanol-*d*_4_) *δ* 8.61 (d, *J* = 2.2 Hz, 1H), 8.53 (d, *J* = 5.3 Hz, 1H), 8.02 (d, *J* = 1.7 Hz, 1H), 7.91 (dd, *J* = 8.7, 2.3 Hz, 1H), 7.55 (dd, *J* = 5.4, 1.8 Hz, 1H), 7.51 (dq, *J* = 8.6, 1.6 Hz, 1H), 7.14 (s, 1H), 6.64 (dd, *J* = 17.0, 10.2 Hz, 1H), 6.47 (dd, *J* = 17.0, 1.7 Hz, 1H), 5.87 (dd, *J* = 10.2, 1.7 Hz, 1H), 3.63 – 3.58 (m, 2H), 2.98 (t, *J* = 7.0 Hz, 2H). ^13^C NMR (151 MHz, DMSO-*d*_6_) δ 165.30, 164.17, 155.45, 150.49, 141.45, 140.59, 139.58, 138.62, 131.65, 131.29, 128.98, 128.22, 124.58, 124.54, 122.04, 117.23, 116.23, 114.44, 107.47, 40.59, 22.36.

*N*-(3-(dimethylamino)-5-(4-(4-oxo-4,5,6,7-tetrahydro-1H-pyrrolo[3,2-c]pyridin-2-yl)pyridin-2-yl)phenyl)acrylamide (**12**). Yellow solid. MS (ESI) m/z: 402.43 [M+H]^+^. HRMS m/z (ESI) found 402.1918 (M+H)^+^, C_23_H_24_N_5_O_2_^+^ calcd for 402.1925. ^1^H NMR (400 MHz, DMSO-*d*_6_) *δ* 12.09 (s, 1H), 10.13 (s, 1H), 8.54 (d, *J* = 5.2 Hz, 1H), 8.07 (s, 1H), 7.71 – 7.70 (m, 1H), 7.57 (dd, *J* = 5.2, 1.7 Hz, 1H), 7.29 (s, 1H), 7.18 (s, 1H), 7.11 (d, *J* = 2.1 Hz, 1H), 7.05 (s, 1H), 6.53 – 6.42 (m, 1H), 6.26 (dd, *J* = 16.9, 2.1 Hz, 1H), 5.74 (dd, *J* = 9.8, 2.3 Hz, 1H), 3.45 – 3.39 (m, 2H), 2.99 (s, 6H), 2.87 (t, *J* = 6.7 Hz, 2H).

*N*-(2-(dimethylamino)-5-(4-(4-oxo-4,5,6,7-tetrahydro-1H-pyrrolo[3,2-c]pyridin-2-yl)pyridin-2-yl)phenyl)acrylamide (**13**). Yellow solid. MS (ESI) m/z: 402.36 [M+H]^+^. HRMS m/z (ESI) found 402.1928 (M+H)^+^, C_23_H_24_N_5_O_2_^+^ calcd for 402.1925. ^1^H NMR (400 MHz, DMSO-*d*_6_) *δ* 12.02 (s, 1H), 9.45 (s, 1H), 8.60 (s, 1H), 8.52 (d, *J* = 5.2 Hz, 1H), 8.08 (s, 1H), 7.90 (dd, *J* = 8.5, 2.2 Hz, 1H), 7.52 (dd, *J* = 5.3, 1.7 Hz, 1H), 7.21 (d, *J* = 8.5 Hz, 1H), 7.11 (d, *J* = 2.4 Hz, 1H), 7.06 (d, *J* = 2.5 Hz, 1H), 6.80 – 6.69 (m, 1H), 6.29 (dd, *J* = 17.0, 2.1 Hz, 1H), 5.75 (dd, *J* = 10.1, 2.1 Hz, 1H), 3.43 (td, *J* = 6.9, 2.5 Hz, 2H), 2.88 (t, *J* = 6.8 Hz, 2H), 2.71 (s, 6H).

**Spectrums**

^1^H-NMR of compound **2**


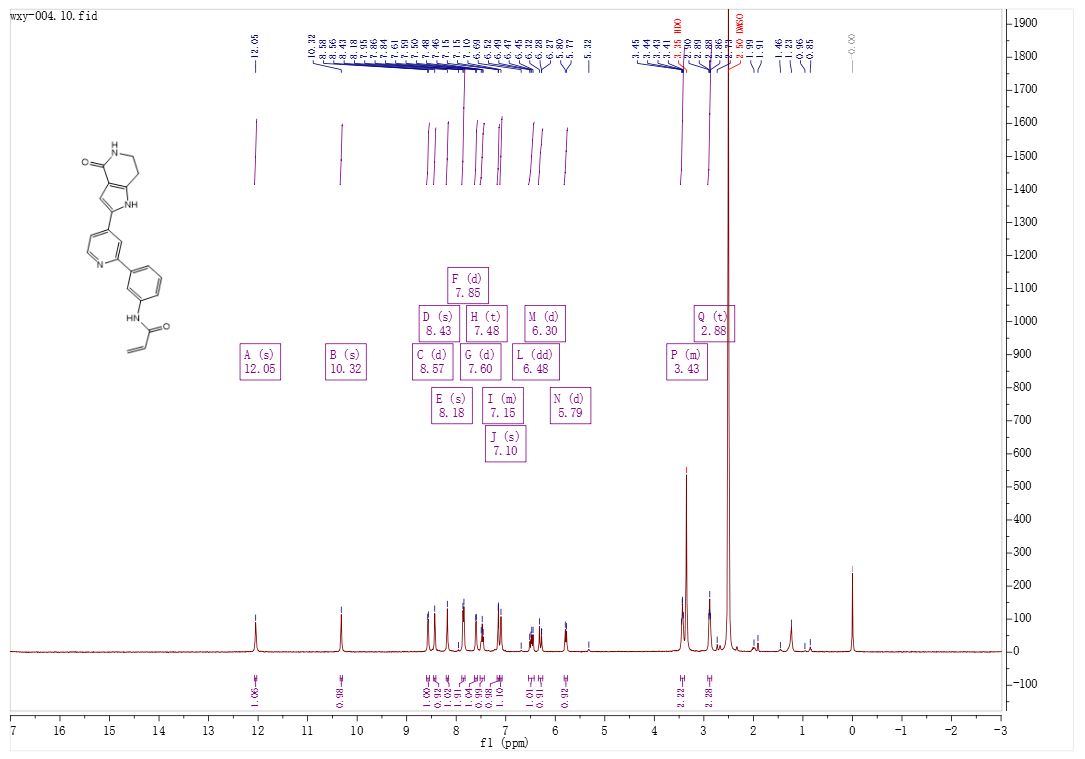


MS of compound **2**


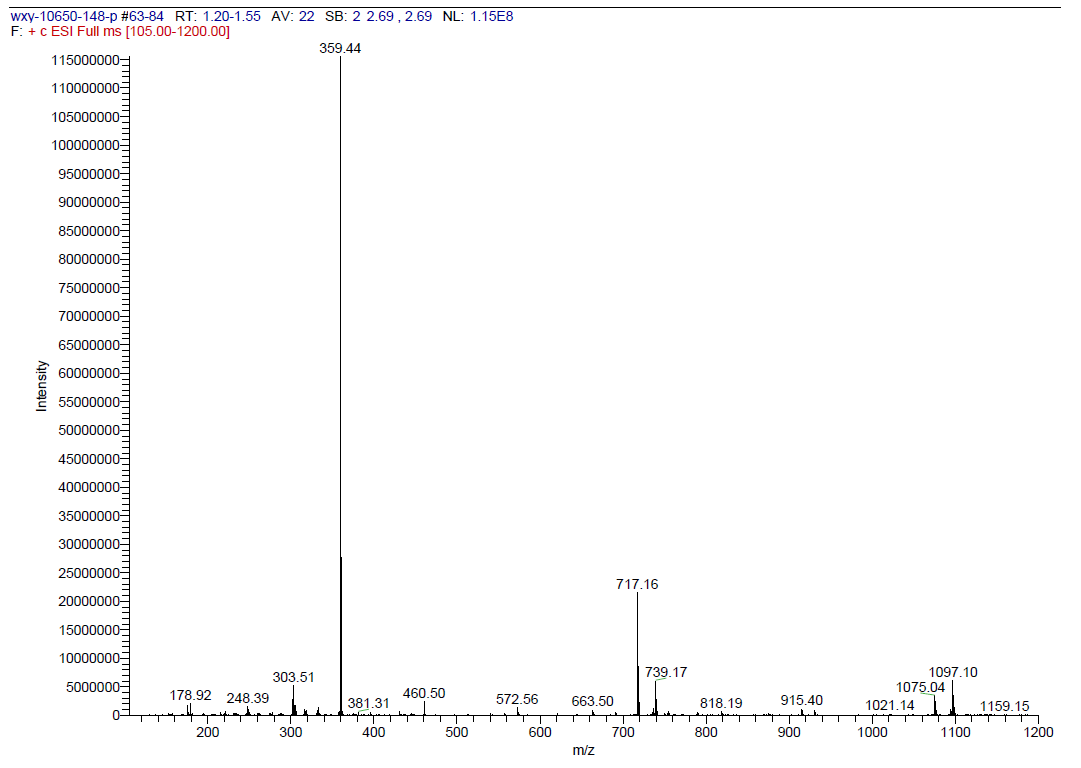


MS of compound **3**


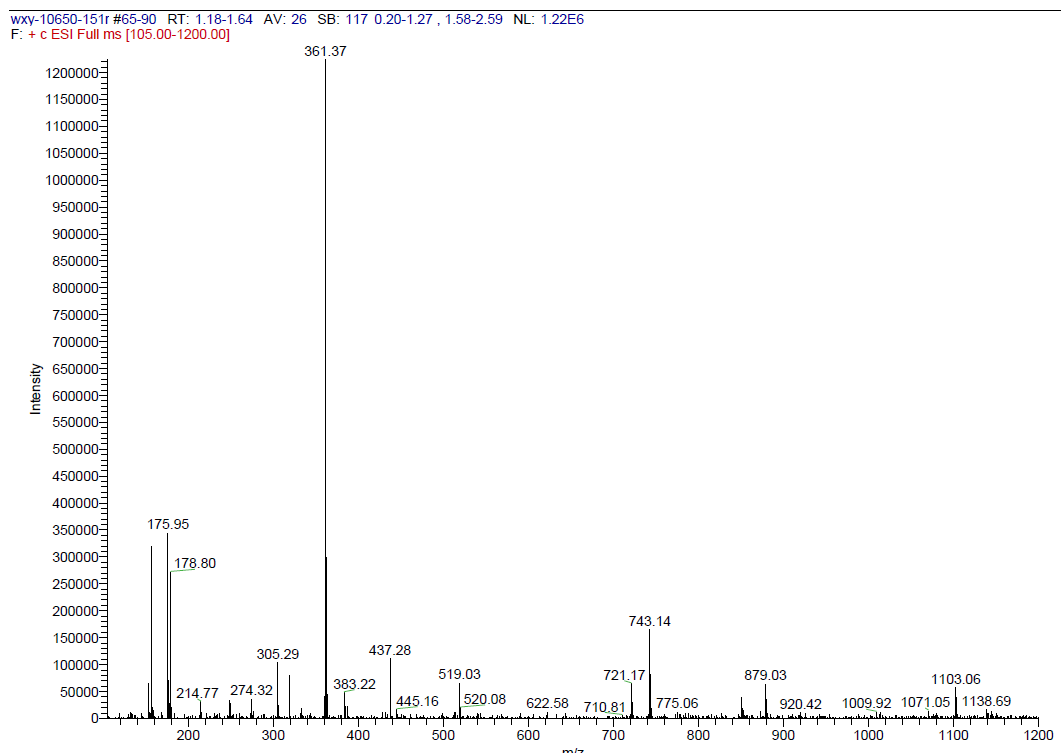


^1^H-NMR of compound **5**


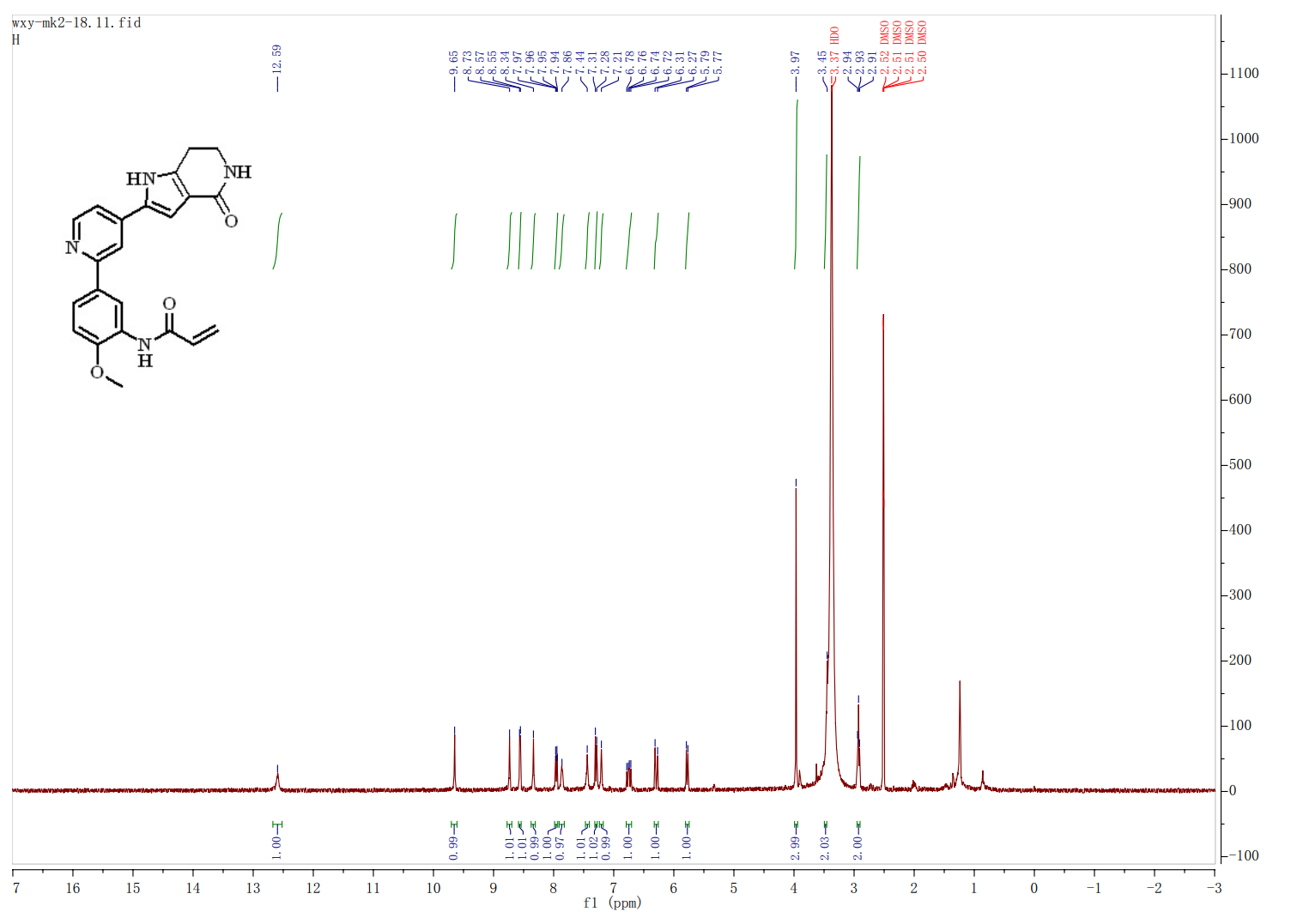


MS of compound **5**


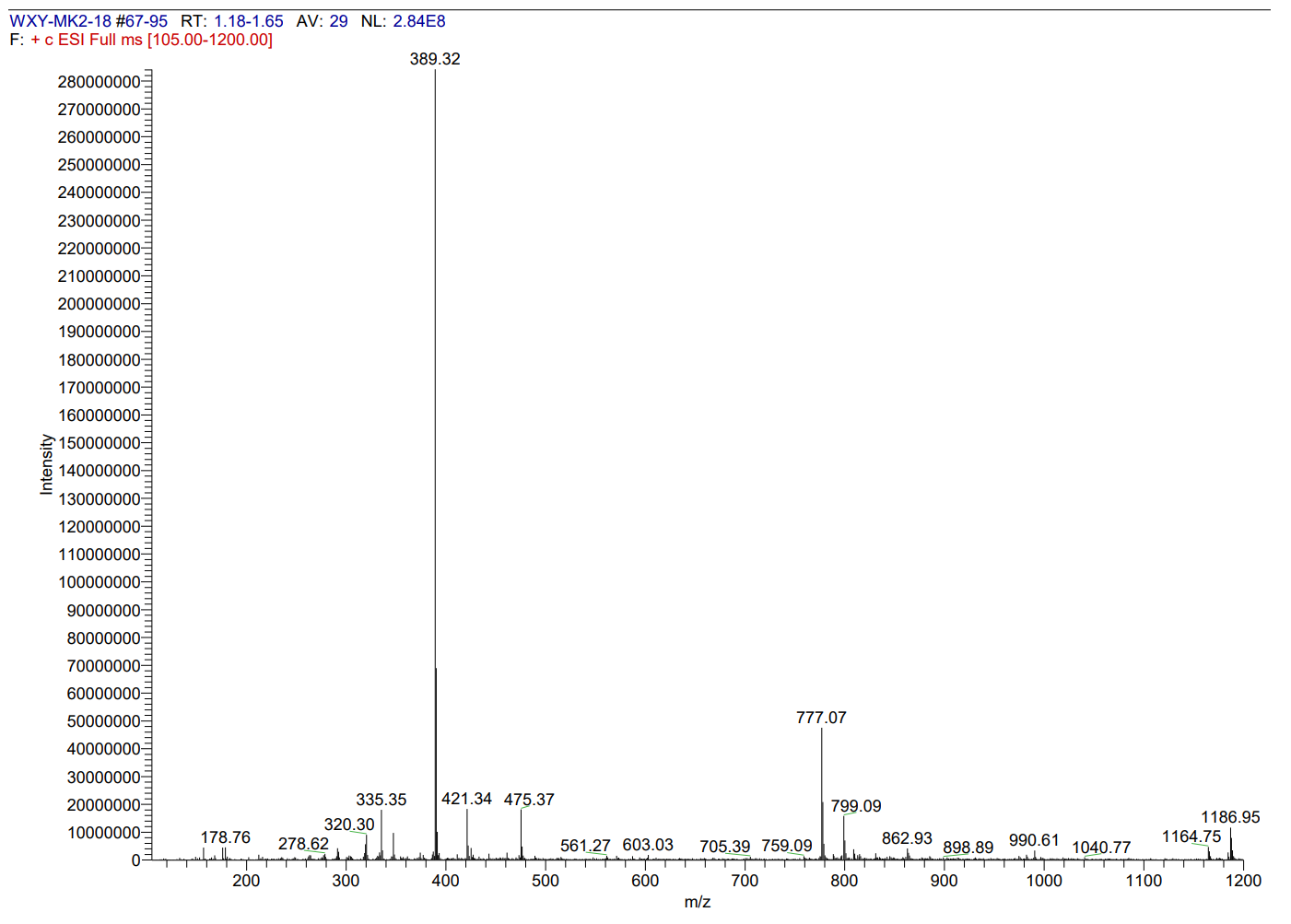


^1^H-NMR of compound **6**


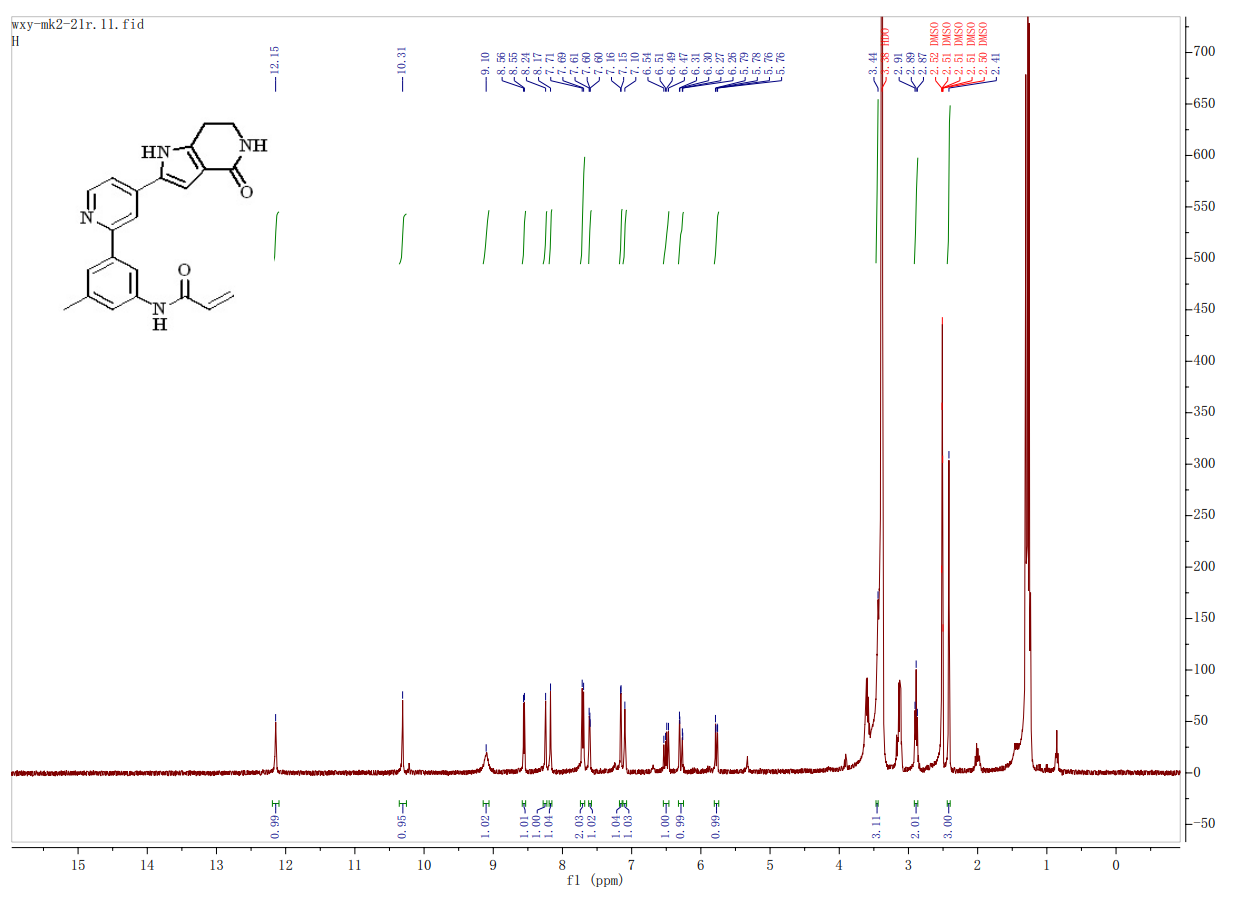


MS of compound **6**


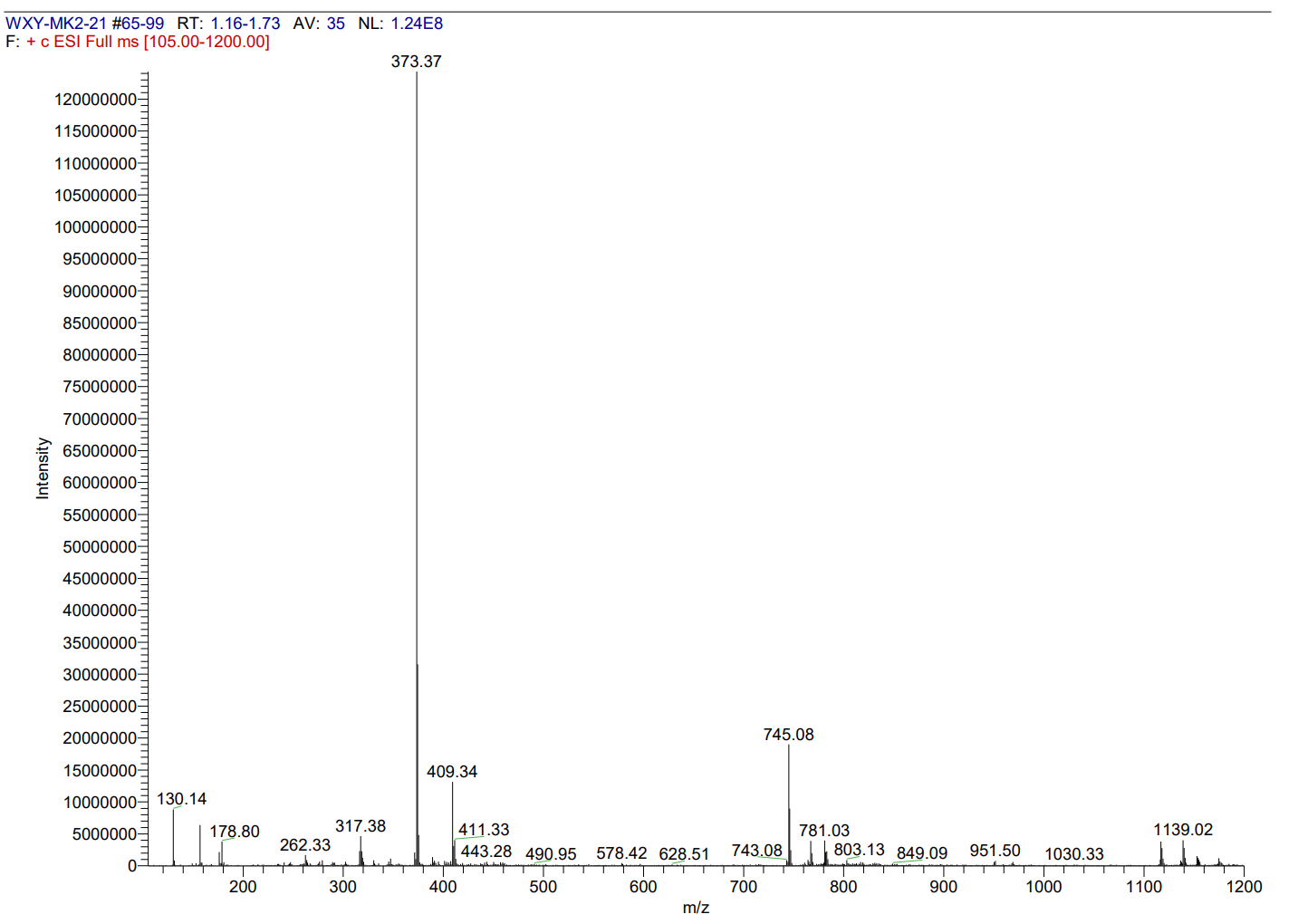


^1^H-NMR of compound **7**


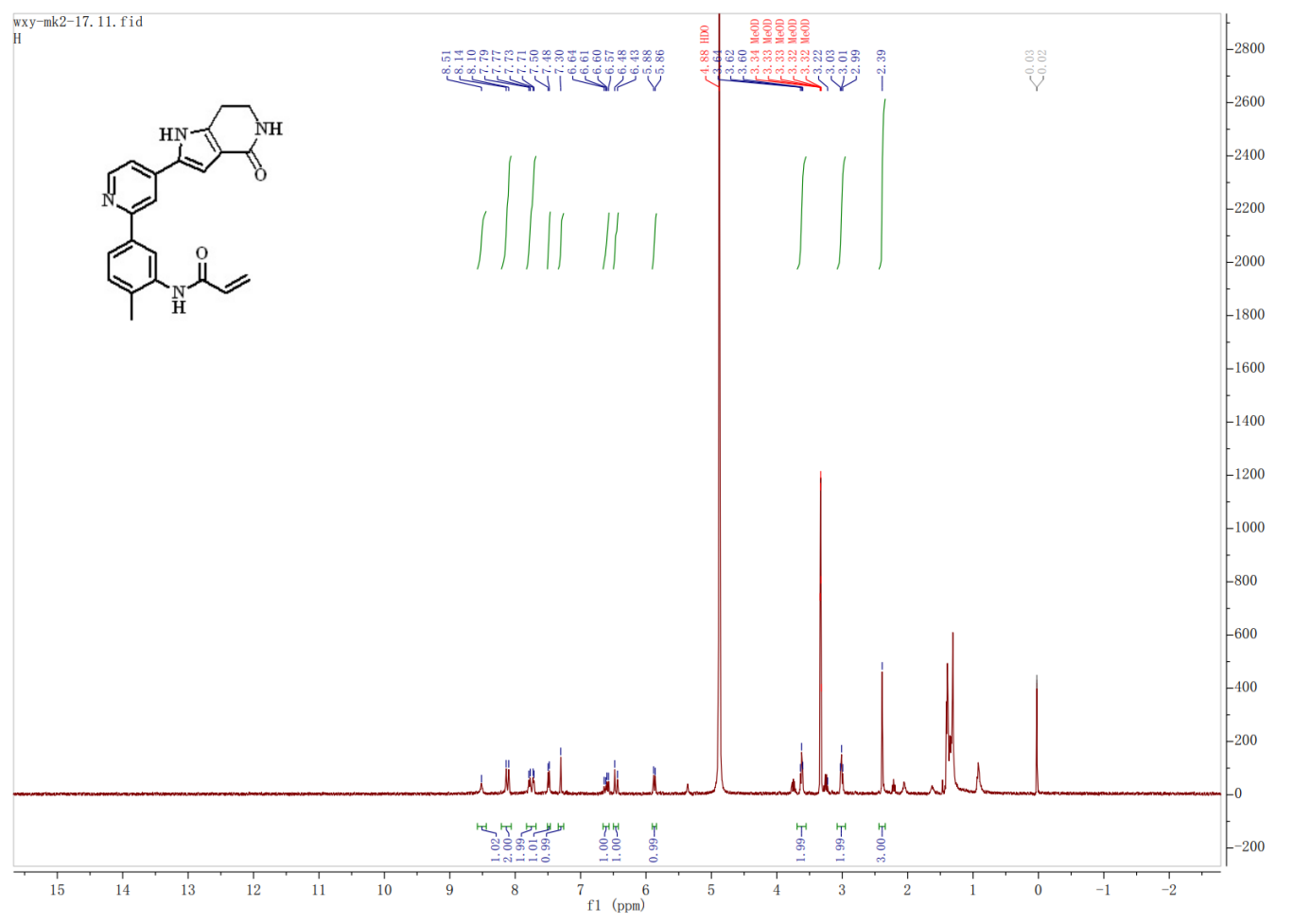


MS of compound **7**


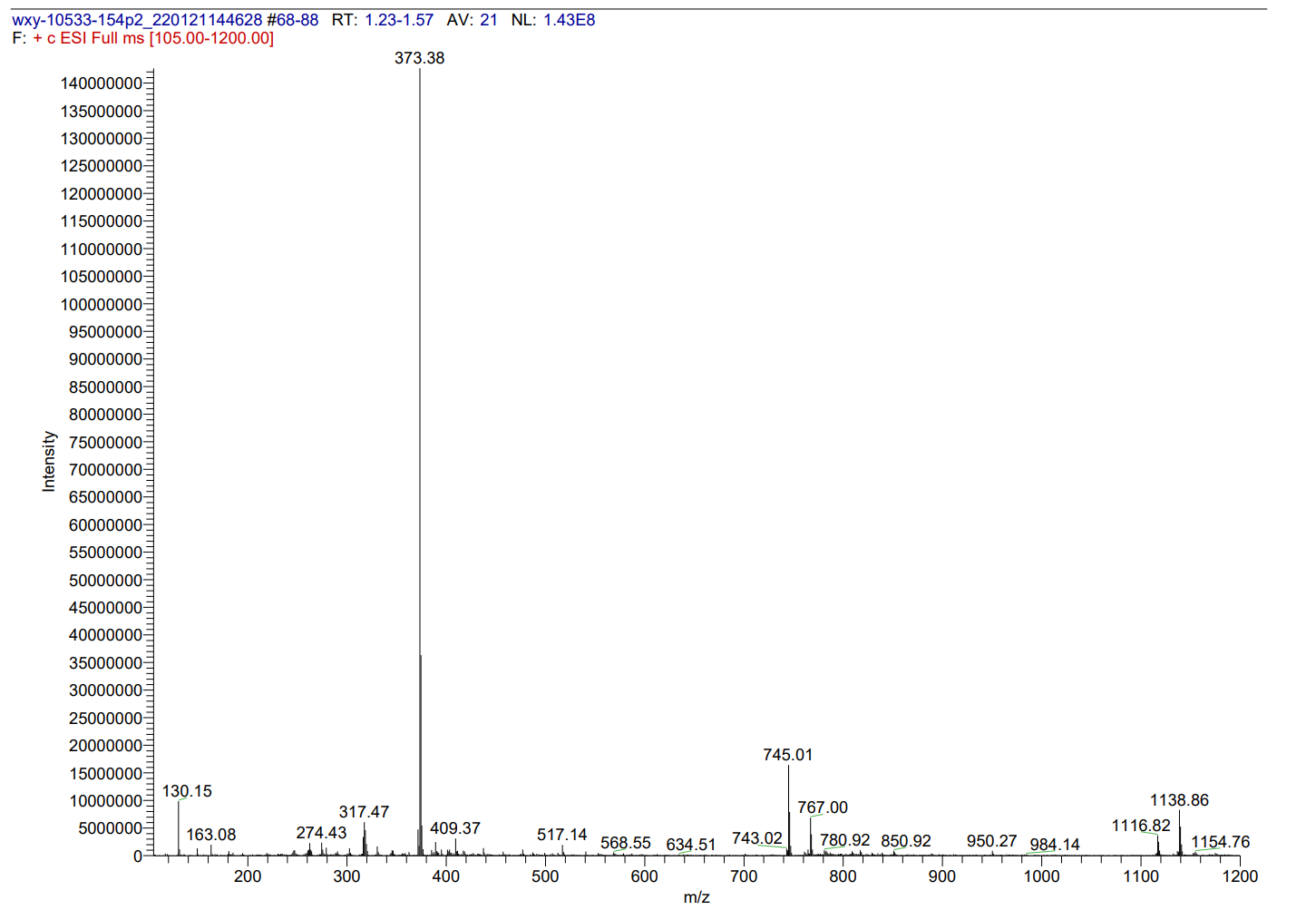


^1^H-NMR of compound **8**


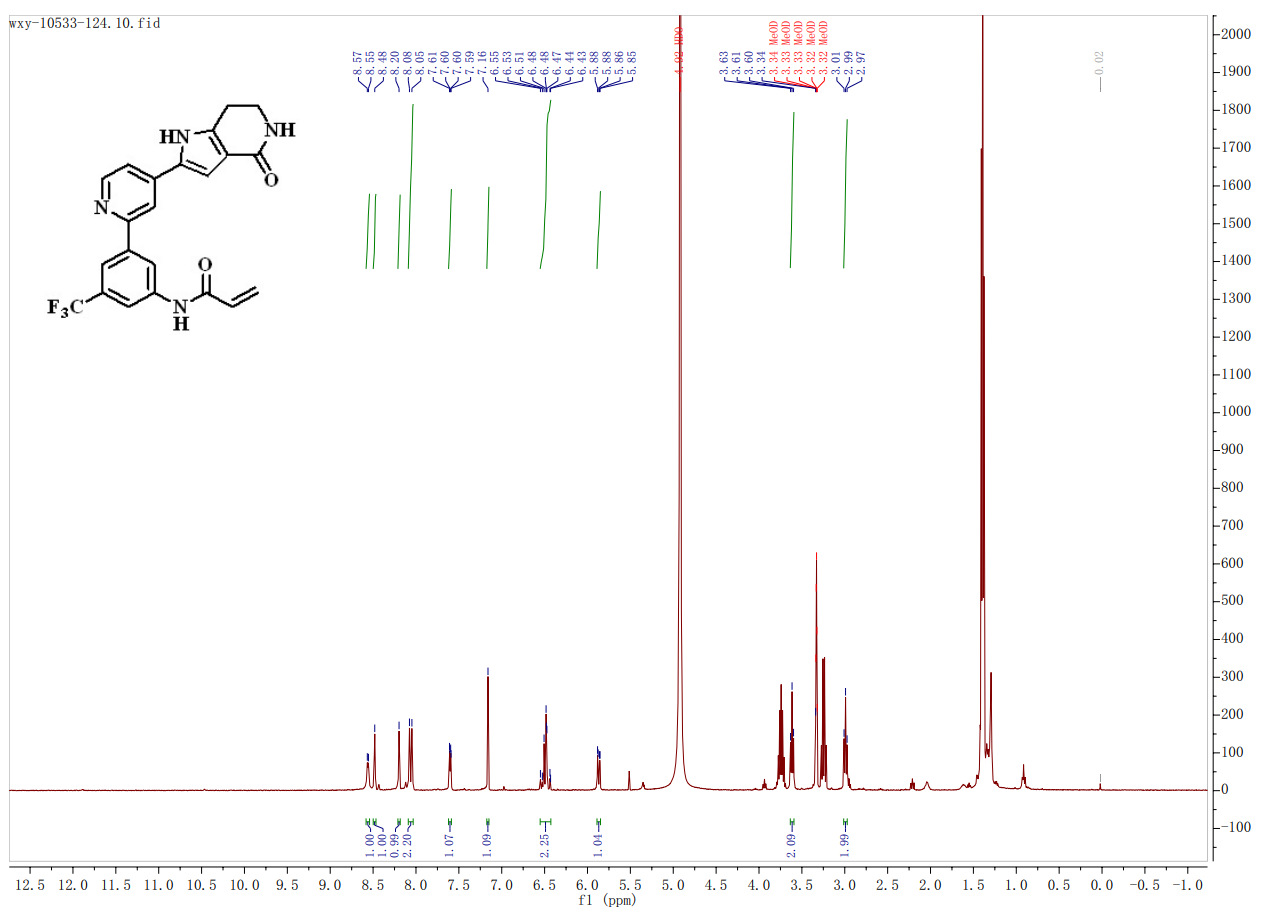


MS of compound **8**


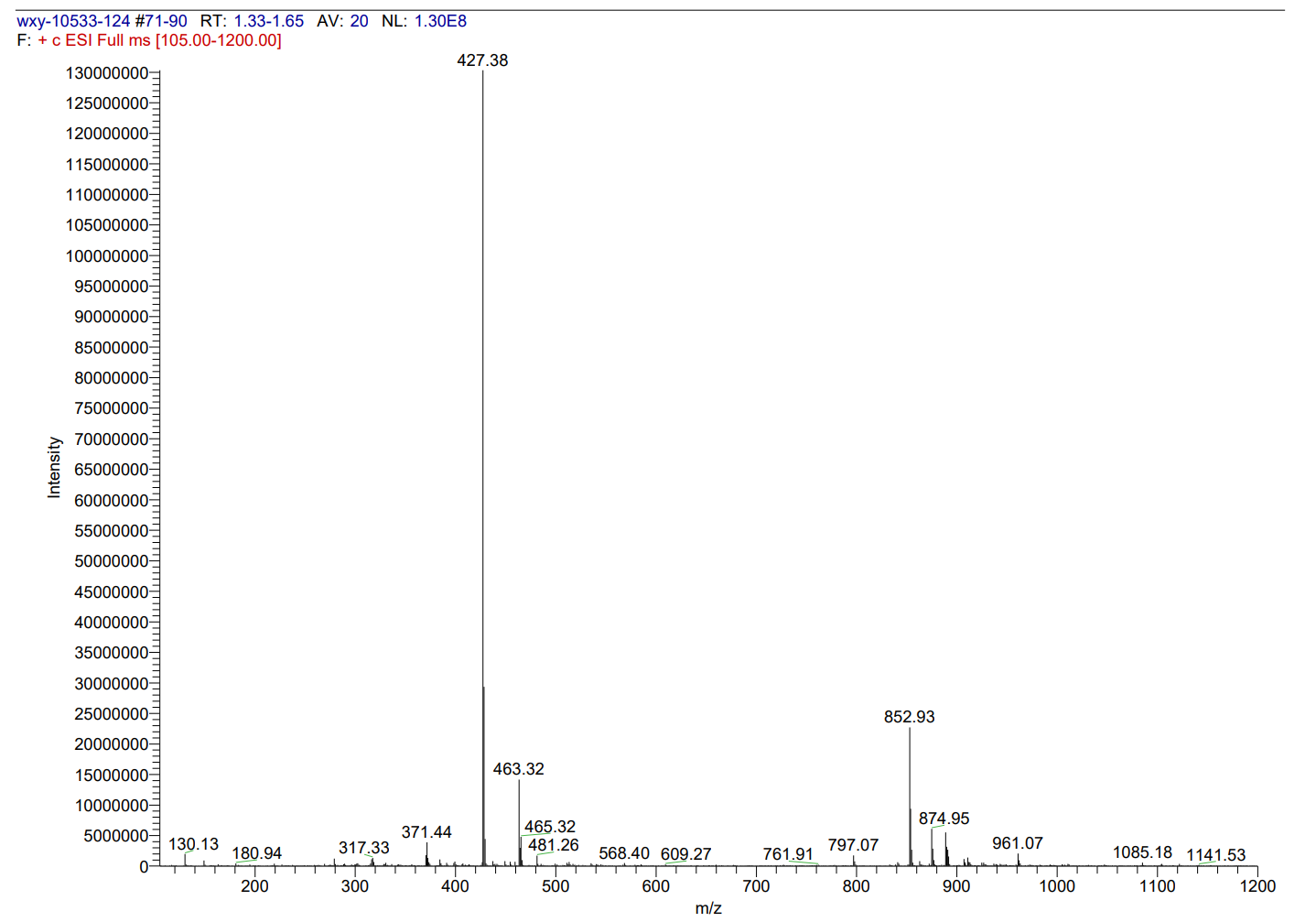


^13^C-NMR of compound **10**

MS of compound **10**


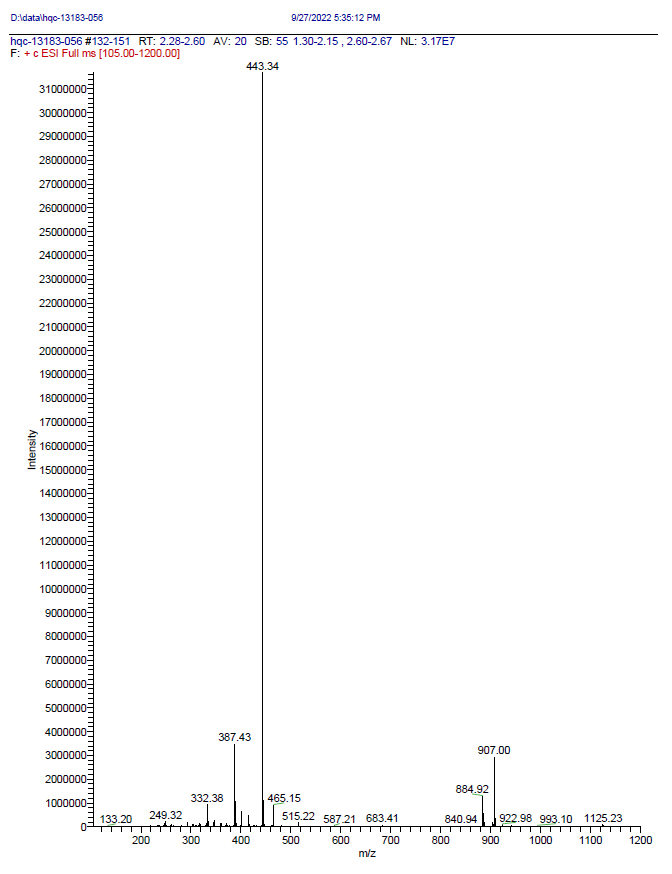


^1^H-NMR of compound **11**


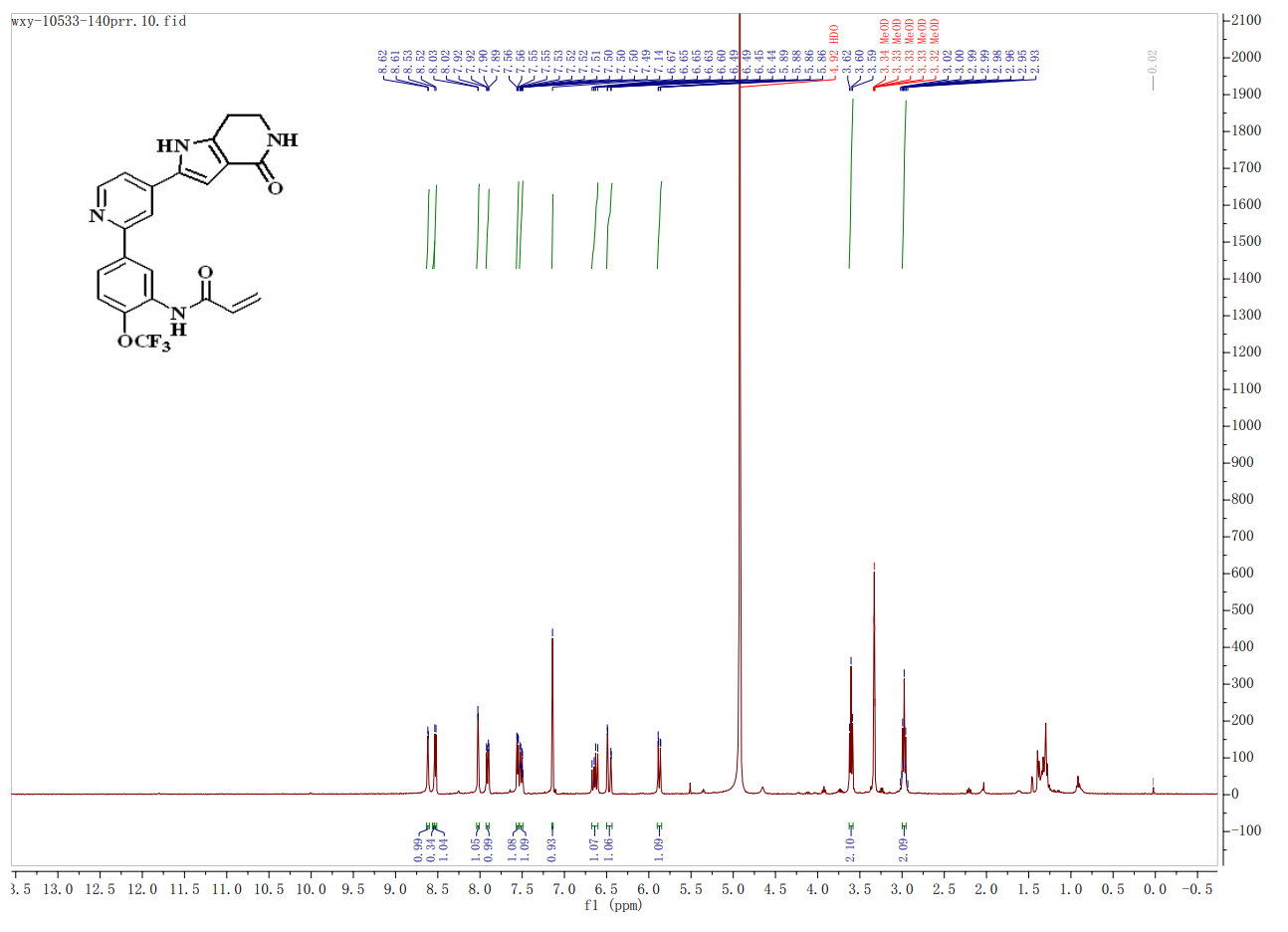


^13^C-NMR of compound **11**

MS of compound **11**


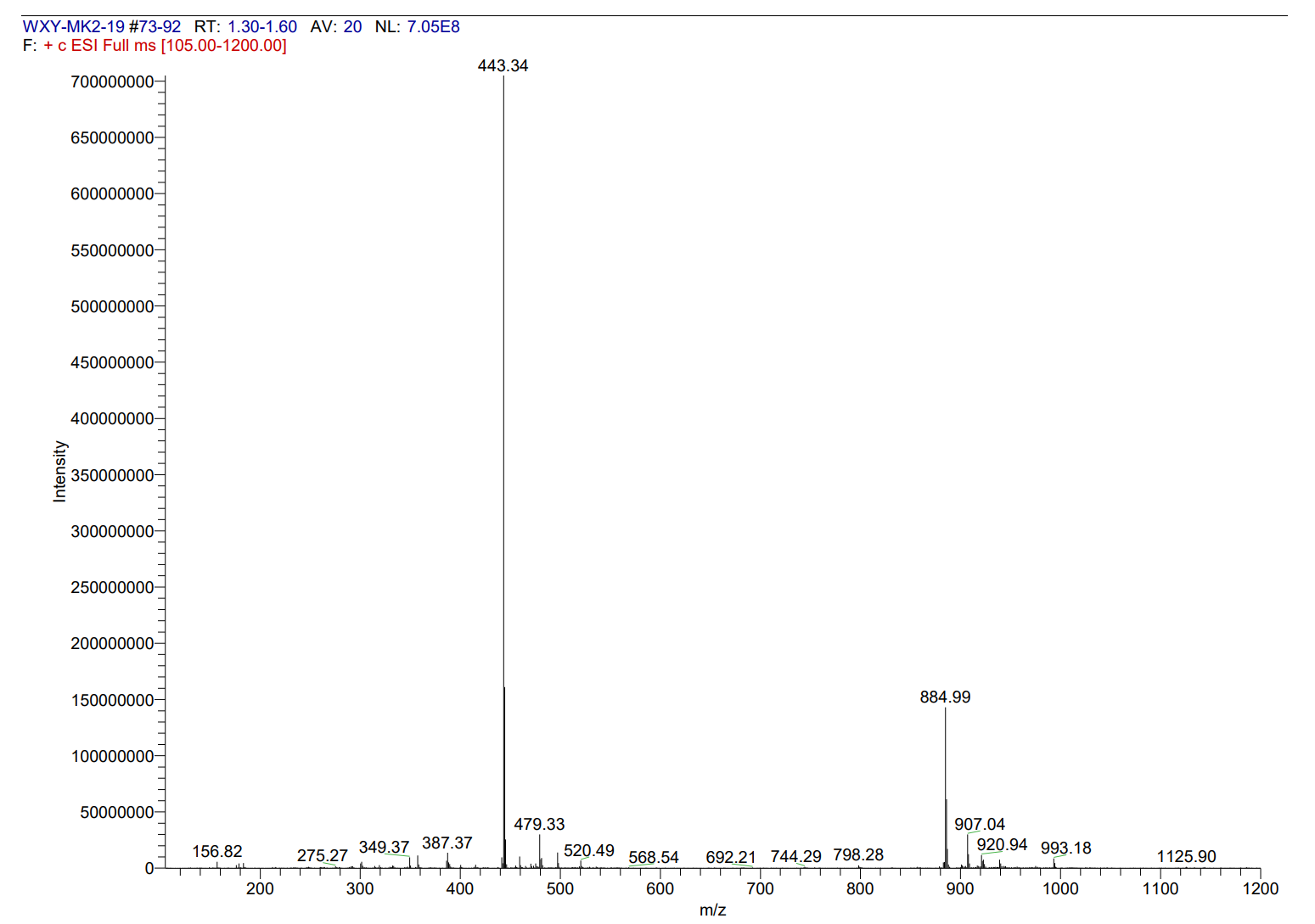


MS of compound **12**


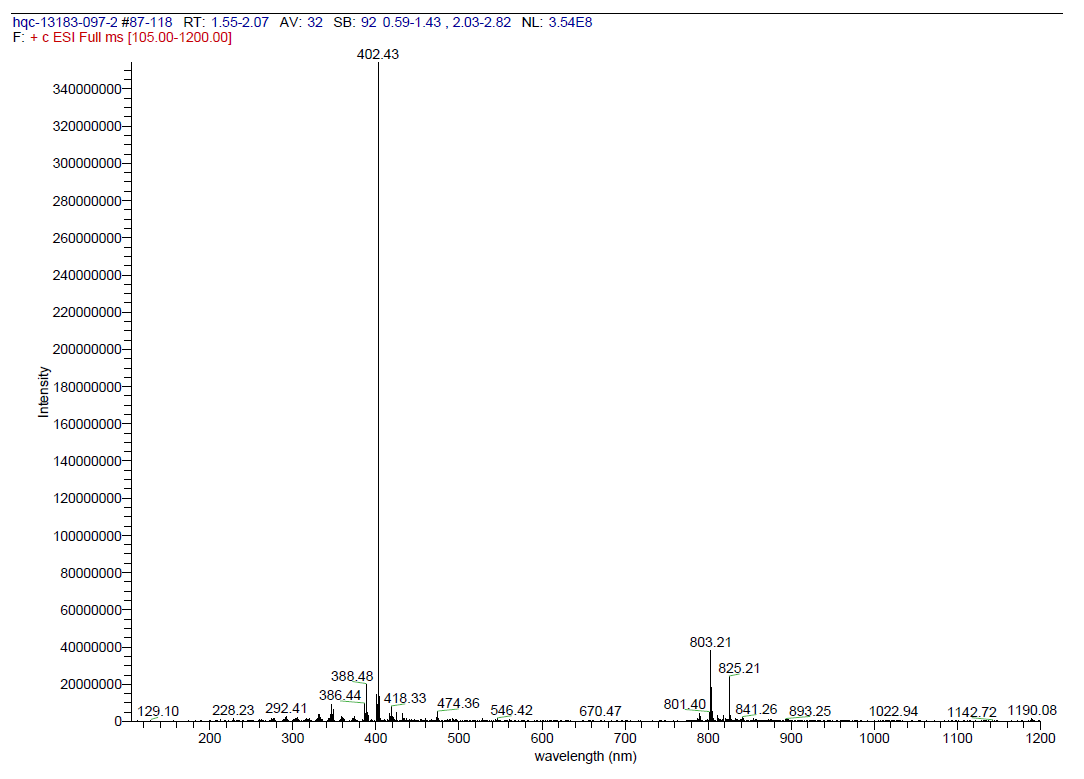


MS of compound **13**


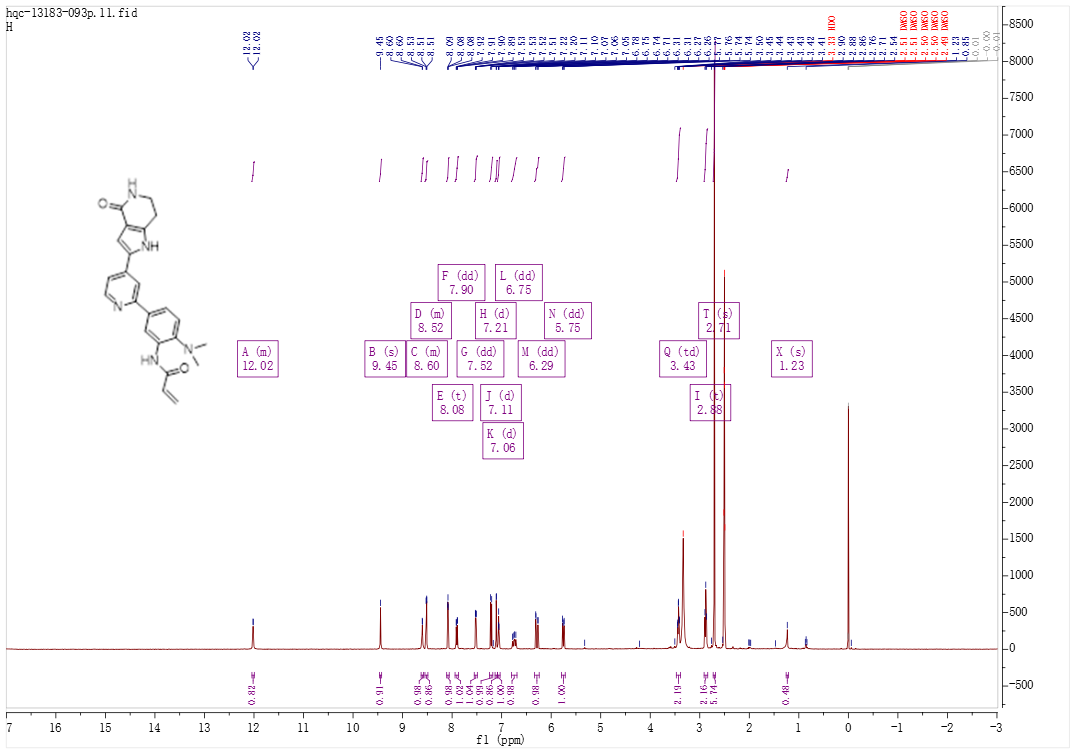


MS of compound **13**


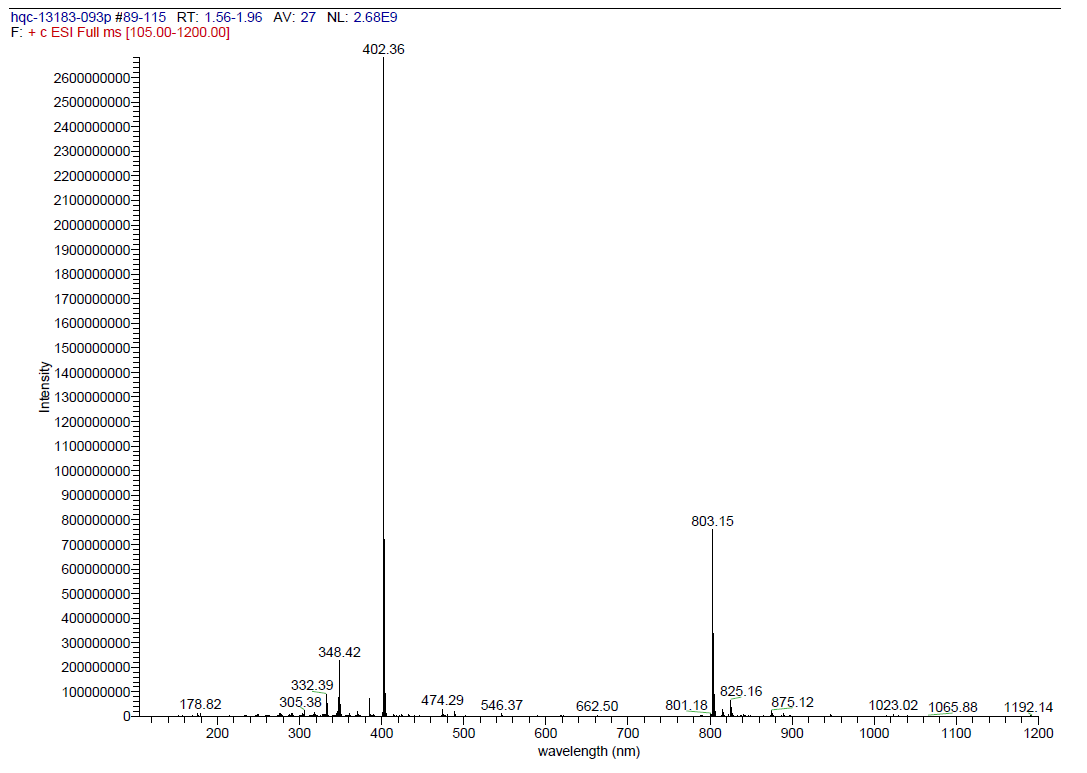

Supplement: Supplementary file 1 — Supporting Information [file MCO2-5-e634-s001.docx]
